# Extensive circadian and light regulation of the transcriptome in the malaria mosquito *Anopheles gambiae*

**DOI:** 10.1186/1471-2164-14-218

**Published:** 2013-04-03

**Authors:** Samuel SC Rund, James E Gentile, Giles E Duffield

**Affiliations:** 1 Department of Biological Sciences and Eck Institute for Global Health, Galvin Life Science Center, University of Notre Dame, Notre Dame IN 46556, USA; 2 Department of Computer Science and Engineering, Fitzpatrick Hall, University of Notre Dame, Notre Dame IN 46556, USA

## Abstract

**Background:**

Mosquitoes exhibit 24 hr rhythms in flight activity, feeding, reproduction and development. To better understand the molecular basis for these rhythms in the nocturnal malaria vector *Anopheles gambiae*, we have utilized microarray analysis on time-of-day specific collections of mosquitoes over 48 hr to explore the coregulation of gene expression rhythms by the circadian clock and light, and compare these with the 24 hr rhythmic gene expression in the diurnal *Aedes aegypti* dengue vector mosquito.

**Results:**

In time courses from *An. gambiae* head and body collected under light:dark cycle (LD) and constant dark (DD) conditions, we applied three algorithms that detect sinusoidal patterns and an algorithm that detects spikes in expression. This revealed across four experimental conditions 393 probes newly scored as rhythmic. These genes correspond to functions such as metabolic detoxification, immunity and nutrient sensing. This includes glutathione S-transferase *GSTE5*, whose expression pattern and chromosomal location are shared with other genes, suggesting shared chromosomal regulation; and pulsatile expression of the gene encoding CYP6M2, a cytochrome P450 that metabolizes pyrethroid insecticides. We explored the interaction of light and the circadian clock and highlight the regulation of odorant binding proteins (OBPs), important components of the olfactory system. We reveal that OBPs have unique expression patterns as mosquitoes make the transition from LD to DD conditions. We compared rhythmic expression between *An. gambiae* and *Ae. aegypti* heads collected under LD conditions using a single cosine fitting algorithm, and report distinct similarities and differences in the temporal regulation of genes involved in tRNA priming, the vesicular-type ATPase, olfaction and vision between the two species.

**Conclusions:**

These data build on our previous analyses of time-of-day specific regulation of the *An. gambiae* transcriptome to reveal additional rhythmic genes, an improved understanding of the co-regulation of rhythms in gene expression by the circadian clock and by light, and an understanding of the time-of-day specific regulation of some of these rhythmic processes in comparison with a different species of mosquito. Improved understanding of biological timing at the molecular level that underlies key physiological aspects of mosquito vectors may prove to be important to successful implementation of established and novel insect control methods.

## Background

The mosquito *An. gambiae* is the primary African malaria vector, whilst *Ae. aegypti* is the primary vector of dengue fever and yellow fever. Mosquito physiology and behavior are under rhythmic control, organized in a time-of-day specific manner. Eukaryotic organisms possess a circadian (“about a day”) clock, regulating daily rhythms in biochemistry, physiology and behavior. It is cell autonomous, and at the molecular level is comprised of a series of transcriptional-translational feedback loops (TTFLs), whose completion takes approximately 24 hr [1]. In *An. gambiae* daily behavioral rhythms are known to include dusk mating swarms, nocturnal flight activity, sugar feeding, blood feeding and oviposition. Late day larval-pupal ecdysis and late day/early night eclosion are also rhythmic [2-14].

*Ae. aegypti* behavioral rhythms have been described from populations collected or observed in the field from around the world as diurnal (often with increased activity during the first and last few hours of the daytime, i.e. crepuscular). These diel/circadian rhythms include flight activity, oviposition, host seeking, human landing/biting and sugar feeding [14-27].

The role of specific *An. gambiae* clock genes in the light-inhibition of blood feeding behavior was revealed by DNA microarray analysis and RNAi-mediated gene silencing [10]. Studies of the mosquito canonical clock components include the cloning of the *Ae. aegypti timeless *gene (*tim,* AAEL006411) [28]; brain *in situ* hybridization of *Ae. aegypti cycle* (*cyc*, AAEL002049) [29]; the expression profiling of clock genes in *Ae. aegypti, An. gambiae,* and *Culex quinquefasciatus*[24,28,30]; the functional analysis of the cytochrome proteins, CRY1 (AGAP001958) and CRY2 (AGAP004261) in *An. gambiae*[31,32]; and geographic and developmental variations in expression of *timeless* in the pitcher plant mosquito, *Wyeomyia smithii*[33].

Recently, we reported in Rund *et al.* genome-wide profiling of rhythmic gene expression in female mated but non-blood-fed *An. gambiae* heads and bodies under both LD (light:dark cycle, 11 hr full light, 11 hr darkness, and 1 hr dawn and dusk transitions) and DD (constant dark) conditions [30]. This work revealed genes involved in processes such as immune response, detoxification, transcription, oxidation/phosphorylation, translation, fatty acid metabolism, glycolysis/gluconeogenesis, olfaction, visual transduction and cuticle-related genes to be rhythmically expressed in *An. gambiae.* Under LD conditions, this included 1293 and 600 rhythmic genes with a period length of 20–28 hr in the head and body, respectively, representing 9.7 and 4.5% of the *An. gambiae* gene set [30]. We studied heads and bodies separately because we expected enrichment (and thus increased detectability) of different genes in the different body segments; for example vision and antennal olfaction-related genes in the head, and genes in the body associated with gut, fat body, and skeletomuscular functions. Under DD conditions, we identified 891 rhythmic transcripts in the head and 476 in the body with an 18.5-26.5 hr period length [30]. A study of *Ae. aegypti* mosquitoes performed by Ptitsyn *et al.*[34]*,* that profiled rhythmic gene expression analysis in the heads of female *Ae. aegypti* mosquito under LD conditions, also revealed transcriptional rhythms in gene expression across a wide variety of biological processes. Our analysis of *An. gambiae* rhythms utilized the COSOPT algorithm to mine expression data, whilst Ptitsyn *et al.,* report results from the Fisher's g-test, autocorrelation and the Pt-test algorithm. The COSOPT cosine-wave fitting algorithm [35-38] is one of several, and arguably the method most used to mine gene expression data for genes rhythmically expressed with a sinusoidal expression pattern [36,37,39-43]. Other methods for identifying sinusoidal expression patterns include the recent JTK_CYCLE algorithm [44-46] and Fourier transform [47-49]. Investigations in maize, mice and artificially generated transcript profiles, for example, have demonstrated differing results in number and identity of genes scored as rhythmic depending on the algorithm used [39,44]. Additionally, there are non-sinusoidal yet still 24 hr patterns of expression, such as pulsatile “spikes” which were noted in maize and *Arabidopsis thaliana* circadian transcriptional analysis using HAYSTACK [39,50], which may be missed by algorithms searching specifically for sinusoidal expression patterns. We note male and female *An. gambiae* mosquitoes have an abrupt onset and short duration of *elevated* flight activity at dusk under both LD and DD conditions [13,30], and therefore we hypothesized this could correspond with “spike” gene expression profiles.

Rhythmic genes exhibiting a 24 hr period length are generated through the intersection of two processes: 1) The first is an endogenous circadian clock that persists under constant environmental light and temperature conditions (true “circadian” expression). The persistence of behavioral, physiological, and/or gene expression rhythms under constant conditions is thus indicative of an endogenous clock. 2) The second is a direct action of the environmental LD cycle on the organism that generates additional diel rhythms (rhythms observed under LD but not necessarily DD conditions) in gene expression and suppresses a proportion of rhythms generated by the endogenous circadian clock mechanism. This direct LD cycle mechanism has been described in *Drosophila* and our *An. gambiae* studies, yet is poorly understood at the molecular level. It presumably includes photoreception, including a contribution from the compound eyes [30,37,48,51].

In this work, we reanalyze our original *An. gambiae* data using the more recently developed JTK_CYCLE algorithm, as well as perform a discrete Fourier transform (DFT) analysis. We use the consensus from these two methods along with our original COSOPT analysis to identify more genes as rhythmic with a high degree of confidence. We use a pattern matching algorithm novel to biological analyses to identify genes displaying clear pulsatile “spikes,” since genes displaying this pattern may be missed by the other algorithms. Next, we further investigated the intersection between light-driven and endogenous clock-driven expression of rhythmic genes by looking at some unique patterns in gene expression that are present as mosquitoes make the transition from LD to DD conditions. We examine the presence of defined transcriptional regulation motifs in the 5' upstream regions (presumed promoter regions) of those genes. Finally, we also reanalyze the *Ae. aegypti* gene expression data of Ptitsyn *et al.* using JTK_CYCLE and compare patterns in ~24 hr rhythmic gene expression in the head under LD conditions between *An. gambiae* and *Ae. aegypti* across a variety of biological functional categories. This is interesting because both species of mosquitoes are vectors of disease, but may show different diel/circadian expression patterns owing to differences in temporal niche (*An. gambiae* is strictly night active and *Ae. aegypti* primarily day active), evolutionary lineage, and/or habitat [52,53]. Improving our understanding of the biology of these vectors (and recognizing the differences between them) is important in generating new methods of control at a time when there is emerging resistance of the mosquito to insecticide and resistance of the malaria parasite to drug treatment [54-56].

## Results and discussion

### Analysis of *An. gambiae* time course data with COSOPT, JTK_CYCLE and discrete Fourier transform reveals new rhythmic probes

Our original analysis [30] of the rhythmic nature of the mosquito transcriptome used very strict criteria to reduce the likelihood of false positives, at the expense of several obvious false negatives. In order to expand this analysis and identify previously unidentified rhythmic transcripts, we reexamined our microarray data to identify novel rhythmic expression patterns at high confidence using an approach of applying multiple algorithms to the same dataset [34,39,47]. We first reanalyzed our microarray data from *An. gambiae*[30], which was originally analyzed using the COSOPT algorithm, using DFT and the more recently developed JTK_CYCLE algorithm. All three of these algorithms search array data for sinusoidal rhythmic expression patterns, but variations in the methods leads to different results. In Additional file 1 we provide the number of probes we identified as rhythmic in each of our four experimental collection conditions (LD heads, DD heads, LD bodies and DD bodies) using various statistical cutoff thresholds. Different cutoff values have been used in various reported studies in an effort to balance the number of rhythmic genes reported against incidents of false positives. In our original COSOPT analysis we used a conservative cutoff of the multiple means corrected β (pMMCβ) of p < 0.1, in an attempt to minimize the occurrences of false-positives. However, in the current analysis we considered probability values as high as p < 0.2 [42,57].

**Figure 1:**
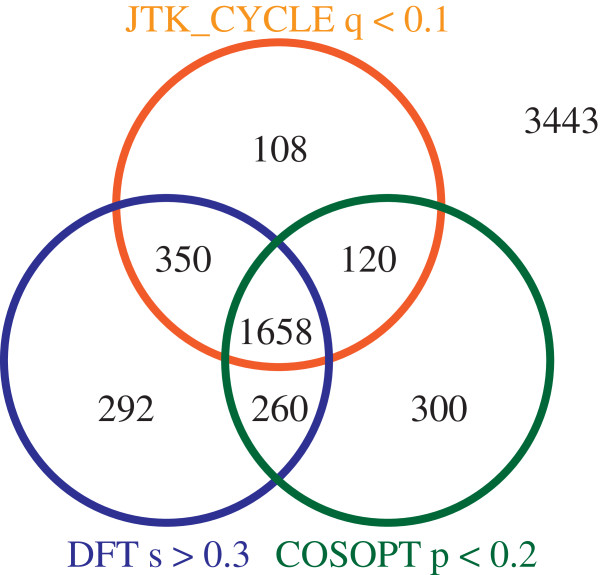
**Analysis of LD head expression data by various algorithms reveals high overlap in *An. gambiae *probes deemed rhythmic.** Venn diagram shows the number of probes in *An. gambiae* LD heads identified as rhythmic using the COSOPT, JTK_CYCLE and DFT algorithms at the statistical cutoffs indicated. A total of 1658 probes were identified as rhythmic using all three algorithms, representing 159 new rhythmic probes from those we identified in Rund *et al.* 2011 [30]. See Additional file 2 for LD body, and DD head and body Venn diagrams. The number outside the Venn diagram, 3443, represents the number of probes with a mean fluorescent intensity above background that were not scored as rhythmic by any of the algorithms. See Additional file 3 for list of probes newly identified as rhythmic.

In heads under LD conditions, when considering the least stringent cutoff values, COSOPT (p < 0.2), JTK cycle (q < 0.1) and DFT (s > 0.3) each returned ~2300 probes determined to be rhythmic. The statistical cutoff values for COSOPT and JTK_CYCLE match the highest thresholds values utilized elsewhere, whilst the DFT value was chosen as it returned approximately the same number of probes [42,44,57]. When we considered the overlap of probes found rhythmic by using each of these three algorithms, 1658 probes were determined to be rhythmic by all three methods (Figure 1). Of these 1658 probes, 159 were not identified as rhythmic using the COSOPT criteria from our previous report [30]. New rhythmic probes were also identified in LD bodies, DD heads and DD bodies, where 148, 47 and 32 probes, respectively, were determined to be rhythmic that were not identified as such in our previous analysis (Additional file 2). Note that DFT analysis limits the number of probes that may be deemed rhythmic under DD conditions; see methods for more information. We believe that these newfound rhythmic genes can be called rhythmic with a high degree of confidence, since three separate algorithms identified them as such. Similar to our previous analysis [30] we found additional rhythmic genes in a range of functional groups dominated by metabolism, but also rich in detoxification, immunity, and cuticular function (see Additional file 3). From the LD head analysis, several of these newly found rhythmic probes reference genes of unknown function, or map to genomic regions not currently identified as genes.

Our reanalysis of microarray data using alternate expression-mining algorithms resulted in the identification of more rhythmic genes that could underlie important rhythmic mosquito physiological processes – notably, detoxification, immunity and nutrient sensing genes. All time course expression profiles, including COSOPT and JTK_CYCLE outputs, can be viewed on our publically accessible database, *Bioclock*[58]. The discovery of more rhythmic genes adds more evidence in *An. gambiae* for rhythmic susceptibility to factors such as insecticide, infection and environmental challenges, as well as targets for manipulation to disrupt important rhythmic mosquito biological processes. Recent work in the closely related mosquito, *Anopheles funestus,* has shown that populations of these important malaria vectors are shifting their biting times in response to the utilization (and therefore selective pressure) of insecticide treated bednets [59]. Future investigations into this phenomenon should consider the current work presented here, as a shift in the expression of one or several of the genes we report as rhythmic might explain or underlie the reported shift in behavior.

#### Detoxification genes newly identified as rhythmic

Detoxification genes newly identified as rhythmic include the glutathione S-transferase (GST), *GSTE5* (AGAP009192), which is noteworthy as it joins *GSTE3* (AGAP009197) and *GSTE2* (AGAP009194), two other GSTs on division 33B of polytene chromosome arm 3R [60] that we previously found rhythmically expressed in LD heads [30]. *GSTE2* is a known resistance gene with a gene product that has been confirmed to metabolize DDT [60]. These three genes share nearly identical times of peak expression, potentially indicating a shared gene regulatory process. Chromosomal regions of rhythmic coregulation have also been noted in *Drosophila*[61]*.* In LD bodies we found five more rhythmically expressed annotated or predicted detoxification genes including *cytochrome P450 6P4* (*CYP6P4*, AGAP002867) and *GSTD11* (AGAP004378) (Additional file 3). All five of these detoxification genes we had previously identified as rhythmic in DD bodies, but not in LD bodies [30].

#### Immunity and nutrient sensing/feeding genes newly identified as rhythmic

Further, the list of genes newly found rhythmic under LD conditions includes components of *An. gambiae* immune gene families including the clip-domain serine protease new to our rhythmic list, *CLIPD5* (AGAP002813, head), and *CLIPE6* (AGAP011785)*,* previously identified as rhythmic in LD heads and now in LD bodies; the class b scavenger receptor, *agSCRB8* (AGAP004845), previously identified as rhythmic in the body but now head; and the serine protease inhibitor (serpin), *SRPN5* (AGAP009221), previously identified as rhythmic in LD and DD heads and now in LD and DD bodies (Additional file 3).

Finally, our previous analysis revealed numerous genes that are involved in nutrient sensing and/or feeding behavior in various conditions/tissues including the *takeout* genes (*TO1*, AGAP004263; *TO2* and/or *TO3*, AGAP012703/AGAP004262), *adipokinetic hormone receptor* (*AKHR*, synonymous with *gonadotropin-releasing hormone receptor, GPRGNR1*, AGAP002156), *target of rapamycin* (*TOR*, AGAP007873), *neuropeptide F* (*NPF*, AGAP004642), and the *Anopheles* homologues to *Drosophila Lipid storage droplet-1* (*LSD1*, AGAP002890), *SNF1A/AMP-activated protein kinase* (*agAMPK,* AGAP002686) and *foraging* (*for,* AGAP008863) [30]. In subsequent work, we revealed time-of-day dependent increases in flight behavior in *An. gambiae* and *Ae. aegypti* by pharmacological activation of the protein kinase G (PKG) encoded by the *for* gene [14]. This is of particular interest as dengue virus infection increases *Ae. aegypti* flight activity behavior [62] and PKG mediates a phosphorylation event involved in dengue virus replication [14].

We now find *agAMPK* (peak phase, ZT 4-ZT 6) and a predicted forkhead domain transcription factor (in *Drosophila, foxo*) (AGAP008606, peak phase ~ ZT 9) additionally rhythmic in the body; new to the rhythmic list, the *Anopheles* homologue to *Drosophila sugarbabe* (*sug*, AGAP006736) was found rhythmic in the body and peaking at the end of the night phase (ZT 22-ZT 0) (Additional file 3). *Drosophila sug* encodes a predicted zinc finger protein that regulates insulin gene expression in neurosecretory cells [63], whilst *Drosophila* FOXO regulates the insulin receptor pathway [64].

### Using a pattern matching algorithm to search for pulsatile expression patterns

**Figure 2:**
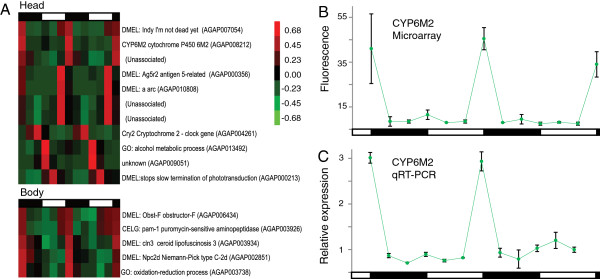
**Pattern matching algorithm reveals genes with pulsatile expression.** A pattern matching algorithm revealed pulsatile expression patterns of 11 probes in LD heads and 5 probes in LD bodies that were rhythmic with a c > 1.6 and peak-to-trough fold change greater than 1.5 (c is the convolution value between probe signals and the pulsatile template). Two of these genes from LD bodies and five from LD heads had not been previously identified as rhythmic under those conditions [30]. (**A**) Hierarchical clustering of genes found rhythmic using the pattern matching algorithm in LD heads (top) and bodies (bottom). Red indicates higher expression, and green indicates lower expression versus the mean value for each gene. (**B**) Gene expression profile from microarray data of one of the new genes found rhythmic in LD heads, *cyptochrome P450 6M2* (*CYP6M2*). (**C**) Quantitative real-time RT-PCR (qRT-PCR) validates microarray analysis gene-expression profile of the pulsatile expression of *CYP6M2* in LD heads. Data are mean ± SD compared with expression of *RPS7*. Day and night are indicated by the horizontal white/black bars.

The COSOPT, JTK_CYCLE and DFT algorithms all search for sinusoidal expression patterns. However, expression of genes that may have a 24 hr rhythmic but non-sinusoidal pattern, and contribute to the rhythmic biology of the organism, may be overlooked by these three algorithms (*i.e.* pulsatile expression patterns). For example, daily onset of flight activity under LD and DD conditions is abrupt and highly elevated [13,30], and we hypothesized that there are phase-coincident pulses (“spikes”) of gene expression associated with such transient behavior. We therefore utilized a pattern matching algorithm to search for expression patterns that were pulsatile, corresponding to spikes in expression with an interval of 24 hr. While we were unable to identify any genes with pulsatile expression under DD conditions (contrary to our hypothesis), we identified 11 genes in the LD heads and 5 in LD bodies with such a pattern (see Figure 2A). Some pulsatile genes were still found to be rhythmic by COSOPT independently, but two of the body genes, a homologue of *Drosophila Npc2d* (AGAP002851) and a putative copper oxidase gene (AGAP003738), were not previously identified as rhythmic [30]. Similarly, in the head we found five genes with pulsatile expression patterns not found as rhythmic in our previous COSOPT analysis [30]. These are a homologue to *Drosophila antigen 5-related* (*Ag5r2*, AGAP000356); a homologue to the *Drosophila* transporter, *I'm not dead yet* (*Indy,* AGAP007054); a *Drosophila* homologue to *arc* (*a*, AGAP010808); a gene predicted to play a role in alcohol metabolism, AGAP013492 (which was also detected as rhythmic in DD bodies); and a gene of unknown function (AGAP009051). Most interesting, however, was the pulsatile expression of *CYP6M2* (AGAP008212) (Figure 2B), a cytochrome P450 monooxygenase that is known to be upregulated in insecticide resistant mosquitoes [65], and has a gene product demonstrated to metabolize pyrethroids [43]. *CYP6M2* was previously identified as rhythmic in *Anopheles* bodies, but not heads [30]. In order to confirm our microarray expression data, we performed qRT-PCR on LD head samples and indeed, confirmed a pulsatile expression pattern of *CYP6M2* (Figure 2C).

### Light regulation of the *An. gambiae* transcriptome

**Figure 3:**
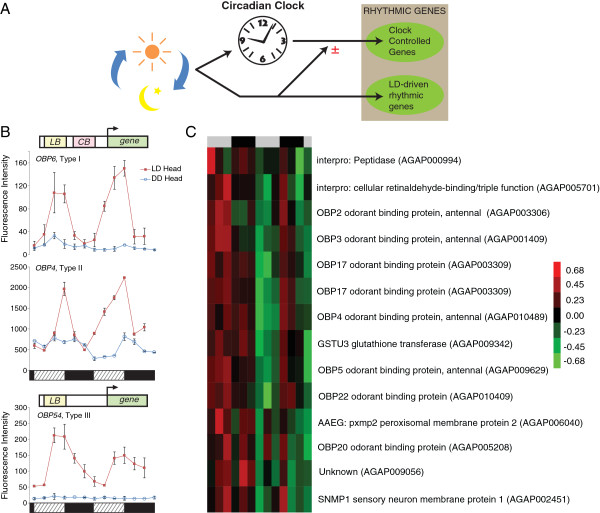
**Light regulation of the *An. gambiae *transcriptome. **(**A**) Model of the regulation of 24 hr rhythmic expression by the endogenous circadian clock and the LD cycle from our studies and other organisms [30,37,48,50,51,69-72]. CCGs are a subset of rhythmic genes with expression driven directly by the circadian clock. Light synchronizes or resets the clock, but activity and rhythms in CCGs persist without LD cycle input. Under LD conditions, additional “LD-driven rhythmic genes” are expressed rhythmically, and a proportion of CCGs have inhibited or enhanced rhythmicity, controlled by both the clock and the direct action of light. (**B**) Microarray data of OBPs highlights the diversity of mosquito light-regulated expression, with various levels of interaction between clock-and light-driven control. Type I group (e.g. *OBP6*) are rhythmic under LD and DD conditions, with amplitude of expression higher under LD conditions. Type II group, (e.g. *OBP4*) have rhythmic expression dampened in DD, but this occurs in the second cycle under constant conditions. Expression in the first cycle does not dampen during subjective day relative to subjective night, as would be expected from LD cycle expression. Type III group (e.g. *OBP54*) has rhythmic expression under LD conditions but virtually no expression under DD. As LD cycle collection began at ZT12, and DD collection at subjective CT0, ZT16 and 20 data are appended to end of the collection. Day/subjective day and night/subjective night indicated by the horizontal hashed/black bars. Hypothesized regulation via light box (LB) and/or clock box (CB) response elements. (**C**) Hierarchical clustering of additional genes clustering with and displaying a similar LD to DD cycle phenomenon as *OBP4* (type II). Expression values normalized to mean value across the time course of each gene, red indicates higher, green lower expression. Subjective day and night indicated by the horizontal gray/black bars. Data shown from head samples.

A significant role of light in regulating gene expression and behavior in the mosquito is becoming apparent [10,30]. In this section, we show that there are a large proportion of *An. gambiae* genes that have expression at least partially driven (or suppressed) by the LD cycle. We show that the odorant binding proteins (OBPs) display three interesting patterns of gene expression as the mosquitoes transition from LD to DD conditions, and propose mechanisms that may underlie the control of gene expression in this subset of genes. For example, rhythmic gene expression can be driven by the LD cycle, the circadian clock, or the interaction of the two (Figure 3A) [30,37,48,51]. Changes in gene expression can be induced in *An. gambiae* through a short light pulse [10], while changes in host seeking or locomotor activity patterns in *Ae. aegypti* and *D. melanogaster*, respectively, can be altered through the modification of light regimes [66,67]. In this section we focus on the mechanism through which the LD cycle may influence rhythmic gene expression. Our previous study revealed more genes to be rhythmic (COSOPT, p < 0.1) under LD conditions than under DD, specifically 1293 versus 891 in heads and 600 versus 476 in bodies, although some genes were rhythmic only under DD conditions [30]. We hypothesized that the difference in the number of genes found rhythmic in the two conditions is primarily the result of a direct action by light in driving (or suppressing) rhythmic gene expression in the mosquito [30] (Figure 3A). At the regulatory level, we propose light box (LB, or light response element) and/or clock box (CB, or clock response element) promoters may drive the rhythmic expression of particular gene(s) [68] (see below for a discussion on this mechanism).

#### Olfactory genes in particular highlight different potential mechanisms of clock- and light-driven gene regulation

*An. gambiae* olfactory genes, and in particular those encoding the OBPs, provided interesting examples of different mechanisms that could underlie rhythmic expression. OBPs are soluble proteins that facilitate the activation of olfactory receptors by transporting odor molecules through the antennal lymph to the receptors in the olfactory membrane [73-75]. Many of these OBP genes we previously found to be rhythmic in the head under LD conditions, peaking around dusk (ZT 12) but not under DD [30] (no additional OPBs were found rhythmic in the new expanded rhythmic list, above). Further inspection, however, revealed three interesting patterns in rhythmic expression exhibited by the olfactory genes as the mosquitoes transitioned from LD to DD conditions (*i.e.* differences in gene expression between a 24 hr day in LD, the first 24 hr day under DD conditions and the second 24 hr day under DD conditions). We define these expression patterns as types I, II and III.

The type I group, *OBP6* (AGAP003530; see Figure 3B)*, OBP7* (AGAP001556)*, OBP14* (AGAP002905) and *OBP26* (AGAP012321)*,* showed rhythmic expression under LD and DD conditions, but with dramatic reduction in expression under DD conditions versus LD conditions. In these genes, expression under DD conditions in the first cycle (24 hr period) was similar to the second cycle (next 24 hr period), with expression increasing during subjective day and falling during subjective night. These two observations suggest that expression of these genes is driven by the action of the circadian clock and the LD cycle through clock boxes and light boxes working in concert. The Clock Box (CB) is a *cis*-acting site that is essential for rhythmicity, whereas the Light Box (LB) mediates most of the light-induced regulation [68].

The type II group contained *OBP2* (AGAP003306)*, OBP3* (AGAP001409)*, OBP4* (AGAP010489; see Figure 3B)*, OBP5* (AGAP009629)*, OBP17* (AGAP003309) and *OBP22* (AGAP010409)*.* The expression levels of these genes is similar to the type I group with its dramatically reduced expression in DD versus LD; however, in the LD to DD cycle transition, expression of these type II genes does not dampen during subjective day (circadian time, CT 0 – CT 12) under the first cycle in DD relative to subsequent cycles (Figure 3B). From this, we can deduce that these genes are all presumably under control of both a CB and a LB that act in concert to drive rhythmic expression at higher amplitude than by the clock alone. Under LD conditions, the clock and light work together to drive robust, high amplitude rhythms in expression. As the mosquitoes transition from LD to DD, there is an initial transition cycle in DD where there is still dependency on inputs from the LD cycle and thus the genes display irregular expression patterns. Finally, in subsequent cycles in DD, rhythmic expression is driven entirely by the clock. To see if other genes might have similar expression patterns, we performed hierarchical cluster analysis of DD head expression on the subset of probes identified as rhythmic under LD conditions (in the expanded list, above) to search for additional genes with similar expression patterns as these type II OBPs. We found 13 genes (14 probes) with similar expression including those for the olfaction gene, *sensory neuron membrane protein 1* (*SNMP1*, AGAP002451) [76] and the detoxification gene, *glutathione transferase U3* (*GSTU3,* AGAP009342) [77] (Figure 3C). All of the clustered genes showed a lower level of expression in DD in the same manner as the type II group of OBPs. This pattern of expression under DD conditions suggests that these 13 genes are under control of both a CB and a LB. Indeed, 5 of these genes, the olfaction genes *OBP7, OBP22, OBP26* and *SNMP1*, and the immunity gene, *galectin* 3 (*GALE3,* AGAP004934), have previously been shown to be down-regulated in the head following acute light treatment presented during late night [10,78].

The type III group of genes, *OBP51* (AGAP006077)*, OBP29* (AGAP012331)*, OBP47* (AGAP007287)*, OBP54* (AGAP006080, see Figure 3B) and *OBP57* (AGAP011368), are rhythmic only under LD conditions. Under DD conditions we see these genes are expressed at or below the nadir level of expression observed under LD conditions. We predict that rhythmic expression of these genes would be driven exclusively by a LB, with no contribution from the circadian clock.

For *OBP6* (type I) and *OBP3* (type II)*,* we confirmed using qRT-PCR a reduction in expression in DD as compared to LD conditions. In mosquitoes studied concurrently under different lighting conditions, expression under DD conditions at CT 12 was found to be at 23 ± 5% and 27 ± 34 % (mean ± SD) of expression levels under LD conditions at ZT 12 (Additional file 4A). Furthermore, when we look at the mean expression level across 44 hrs of genes rhythmic under LD conditions (in the expanded list, above), we find that while most probes showed nearly identical expression between LD and DD heads, significant variation between LD and DD expression levels does occur in a smaller subset of genes. The difference in bodies was more pronounced, where 47% of rhythmic body genes show >2-fold differential expression in DD compared with LD (Additional file 4B).

These data reveal a complex interaction between clock-derived signals and photic signals that act on the regulation of OBPs in particular, but also on other genes such as *GSTU3* and *SCRB1*. In fact, specific genes found in all three groups have been previously reported to show reductions in their expression following a light pulse presented during the late night phase of the LD cycle. These include *OBP26* (type I), *OBP22* (type II) and *OBP47* (type III) [10]. Moreover, these gene expression changes are correlated with suppressed feeding behavior, and in fact, manipulation using RNAi knockdown of *OBP4* (type II group) results in altered blood-feeding behavior [10]. Clearly, the current findings are particularly interesting as it highlights the potential for manipulating the mosquito olfactory system, and thus perhaps behavior, through timed light exposure. Indeed, *OBPs 47*, *3*, *7*, *17*, *4* and *22* that we describe here are likely involved in host seeking as they are enriched at least 2-fold higher in female than male antennae [73].

#### The role of light regulation and the molecular circadian clock in rhythm generation

To explore further the effect of light on the regulation of rhythmicity, we also examined in the head the amplitude of the canonical clock components *PER* (AGAP001856), *TIM* (AGAP008288), *CRY2* (AGAP004261), *CYC* (AGAP005655) and *PDP1* (AGAP006376), identified as rhythmically expressed in *An. gambiae* (COSOPT, p < 0.1; JTK_CYCLE, q <0.05) [30]. For *PER*, *TIM* and *CRY2*, we find a consistently smaller peak-to-trough amplitude in the DD compared to LD conditions, a consistent reduction in the JTK_CYCLE algorithm determination of amplitude [44], and a sequential reduction in amplitude between the first and second cycle in DD that is not apparent between cycles in LD conditions (Additional file 5). For *CYC* there was variability between probes in the condition effect, and for *PDP1* rhythm amplitude between conditions was lower. However, no reduction between the first and second cycle in DD was detected. This dampening of the key elements of the transcriptional translational feedback loop (TTFL) of the circadian clock in DD has been observed in *Drosophila*[79-81].

To understand the potential mechanism through which light independently regulates these rhythms in *An. gambiae*, we must turn to genetic model organisms such as *Drosophila*. Genetic deletion of the clock has revealed that some LD rhythms are independent of the circadian pacemaker [48]. Amplitude of output processes does however appear to be coupled to the TTFL of the clock, but evidence for this dependency is mixed [82,83]. For example, the rhythms in *Drosophila* clock proteins PER and TIM, clock controlled gene (CCG) expression and locomotor behavior, do persist even when their corresponding *per* or *tim* gene expression is artificially held constant [84].

It is plausible that the small level of dampening in the rhythms of elements of the TTFL observed in *An. gambiae* within the first two cycles in DD could contribute to changes in CCG expression. However, it is unlikely that it would be the primary cause for the dramatic loss or reduction in rhythmicity observed for many CCGs, such as the *OBP*s.

At least in the rhythms observed in the head, it is likely that the compound and simple eyes contribute to the mechanism of light regulation. In *Drosophila* mutant for the intracellular photoreceptor dCRY (CRY1 in the mosquito), flies are still responsive to light and their LD cycle-driven rhythms persist [48]. However, flies with a mutant phospholipase C component of phototransduction, NORPA (no receptor potential A), have a loss of light regulated rhythms [48]. In the mammalian clock, discrete signaling by light and by the clock is apparent in the regulation of the immediate early genes and/or clock genes *c-fos*, *mPer1* and *mPer2*[85]. Light in this case results in transient gene expression that is associated with resetting of the clock, and light acts indirectly via the Ca^2+^/cAMP response element (CRE). In contrast, the clock components act upon the E box element(s) in the promoter regions of these genes.

At least based on precepts primarily from the *Drosophila* system, we would propose a model for *An. gambiae* to explain our results that consists of: i) separate clock response element(s) or ‘clock box’ (CB) and light response element(s) or ‘light box’ (LB) within the promoters of rhythmic genes; and/or ii) the action of light signaling impinging upon pathways upstream of the CB but downstream of the TTFL. This model is not unreasonable given the complexity of light/circadian regulation being uncovered in genetic model species from several taxonomic groups [48,50,68,82,83,86,87].

#### Clock- and light-regulated response element gene promoter search

In an attempt to identify potential circadian clock- and light- response elements we next searched for promoter elements identified in *Drosophila* as contributing to rhythmic gene expression. Specifically, we searched the 5kb 5' region upstream of the transcription start sites in type I OBPs, type II OBPs and the other genes with similar expression patterns (see Figure 3C), and type III OBPs, for E boxes (from the very generic CANNTG to the canonical CACGTG sequence), W boxes, CREs, Per repeat (PERR) elements, Tim-E-box-like repeat (TER) elements and PDP1 binding sites (PDP1s) [49,88-95]. We find that all 22 genes show examples of at least two different consensus sequences within their upstream region (Additional file 6). We find the occurrence of one or more TER sequences in the upstream regions of all genes except for *OBP14* and *OBP57* (which we note both have upstream regions of <1.8 kb). W boxes and CREs also appear well represented across all groups with at least one occurrence in 12 and 9 upstream gene regions, respectively. We note no PERRs or PDP1s were found in any type III OBPs. These promoter sequences are considered to be definitive clock regulatory elements [91,94,95]. PERR elements were found only in type II genes, with 3 examples of gene upstream regions that have at least one occurrence. PDP1s appeared in 2 of 4 type I upstream regions and 6 of 13 type II regions. Surprisingly, the presence of consensus sequences implicated in clock-regulation including W boxes, TER elements and canonical E boxes, were found extensively in the promoter regions of type III genes. Finally, we find that 9 genes from across all types have a least one occurrence of CREs in the upstream promoter regions, which is not surprising as all type I, II and III genes appear to be at least partially regulated by the direct action of the LD cycle. CREs in mammals are critical to transducing light information to the clock [85], and is plausible that CREs may also contribute to light-regulated expression of the OBPs and other genes in the mosquito.

### Comparisons between rhythmic gene expression in *Ae. aegypti* and *An. gambiae*

Recently, rhythmic expression profiling of the *Ae. aegypti* mosquito was performed in a similar manner to our *An. gambiae* transcriptional profiling [34]. With the publication of these data, we were able to undertake a detailed comparison of rhythmic gene expression between the two species and describe our results in this final section.

Both species of mosquitoes are vectors of disease, but may show different diel/circadian expression patterns owing to differences in temporal niche, evolutionary lineage [52], and/or habitat [53]. *An. gambiae* is strictly nocturnal in its patterns of flight activity, sugar and host seeking, blood feeding, mating, and ovipostion behavior [2-4,7-12,14,30,96-100], whilst *Ae. aegypti* is diurnal, primarily active during the mid-late afternoon (*i.e.* ~ZT 6-12, where ZT 12 is defined as lights off) [14-16,20-25,27,101,102]. If we consider flight activity behavior for example, *An. gambiae* is active throughout the night and rests exclusively during the day, as well as shows a transient elevation of activity at the end of dusk/early night phase, coincident with swarming behavior. *Ae. aegypti* is most active during the latter half of the day/light phase, and tends to show peaks in activity at dawn/early morning and especially so at the end of the day/dusk (i.e. crepuscular); *Ae. aegypti* shows little or no activity during the night. Coincident with flight activity, similar temporal patterns have been shown in the field and laboratory for biting behavior: with *An. gambiae* biting occurring during the night, and *Ae. aegypti* during the morning and late afternoon.

A better understanding of the differences and similarities, and thus potentially different physiological or behavioral responses, in rhythmic gene expression between these two species may prove important in the design and implementation of future control strategies. As an example, we recently demonstrated that when *Ae. aegypti* and *An. gambiae* females were injected with a pharmacological protein kinase G (PKG) activator, 8-pCPT-cGMP (Guanosine-3^′^-5^′^-cyclic Monophosphate, 8-(4-Chlorophenylthio)), both species showed several days of increased flight/wing beat activity, but only at the times of the 24 hr day of their normal flight activity profile when they would normally be active [14].

**Figure 4:**
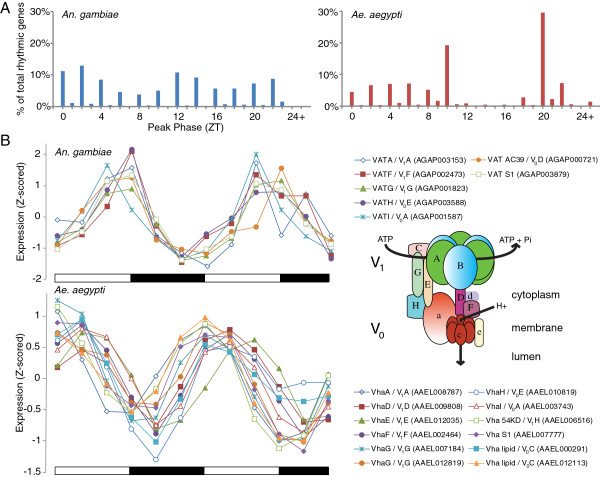
**Timing of gene expression in *An. gambiae *and *Ae. aegypti. ***(**A**) Peaks of transcriptional expression compared between *An. gambiae* and *Ae. aegypti.* Data are binned according to their time value up to and including the phase indicated. Indicated percentages are the proportion of genes that are rhythmic (JTK_CYCLE, q < 0.05) at that peak phase. For genes with multiple rhythmic probes, only the probe with the lowest q value was considered. (**B**) Multiple subunits of the vesicular-type ATPase are rhythmically expressed in both *An. gambiae* and *Ae. aegypti*, but in antiphase. Expression data have been Z-scored. Seven and 10 of the V-ATPase subunit genes are rhythmically expressed and are mostly phase concordant in *An. gambiae* and *Ae. aegypti*, respectively. The peak in expression between the two species, however, are in opposite phases. *Ae. aegypti* subunits and *An. gambiae V-type proton ATPase catalytic subunit A* (*VATA*, AGAP003153) are named in VectorBase. All other genes shown are orthologs predicted using the Database for Annotation, Visualization, and Integrated Discovery (DAVID) [103,104]. As *Anopheles* collection began at dusk (ZT 12) and *Ae. aegypti* collection at dawn (ZT 0), the second and third timepoints from the *Anopheles* collection are appended to the end of the collection as the last two timepoints for visualization purposes. Inset cartoon is a model of V-type H+ ATPase showing the V_1_ and V_0_ complexes. Day and night are indicated by the horizontal white/black bars below the charts. All data shown are from LD heads.

In order to make as similar as possible comparison of rhythmic gene expression between the two species, from experiments of slightly different design, we reanalyzed both datasets using JTK_CYCLE with identical criteria, a stringent q < 0.05 probability cutoff and a period length of 20-28 hr. Interestingly, when we look at the distribution of peak phases (the number of genes which have their peak in expression at any particular time of the day) we find that *An. gambiae* have genes peaking in expression at all times of the 24 hr day, but an enrichment in the number of genes peaking at the dawn and dusk transitions. *Ae. aegypti*, however, has a low percentage of genes with rhythmic expression profiles peaking during ZT 11-17 (first two-thirds of the night phase) (Figure 4A). Interestingly, this is coincident with the nightly *Ae. aegypti* rest period.

Table 1 presents the number of genes from the various biological categories that we found rhythmic in *An. gambiae* (a total of 1400 rhythmic *An. gambiae* genes)*,* the number of those genes where an *Ae. aegypti* homologue is identified in VectorBase (a total of 1202 *An. gambiae* genes had an *Ae. aegypti* homologue), and the number of those 1202 *Ae. aegypti* genes that were rhythmic themselves (a total of 539 genes). See Additional file 7 for details of the 539 common genes. Overall, we confirmed that the *Ae. aegypti* transcriptome is highly rhythmic (4475 genes were identified as rhythmic using JTK_CYCLE), and many genes rhythmic in *An. gambiae* have homologues that are also rhythmic in *Ae. aegypti.*

**Table 1:** **Comparing rhythmic *An. gambiae *genes to *Ae. aegypti *rhythmic genes**

**Category**	**Rhythmic *Anopheles *genes**	***Aedes *homologues**	**Rhythmic *Aedes *homologues**
Chromatin Modification	19	17	8
Detoxification	38	35	18
Immunity	47	40	25
Metabolism	222	207	99
Neuronal/Behavior	43	39	16
Olfaction	29	28	8
Other	106	103	39
Protein Folding/Modification	43	39	21
Proteolysis	47	42	16
Redox	54	51	24
Signal Transduction	79	72	29
Structural	47	43	18
Transcription	68	63	30
Translation	49	46	15
Transport	123	46	49
Unknown	373	319	120
Vision	13	12	4
**Totals:**	**1400**	**1202**	**539**

The list of *An. gambiae* genes found rhythmic in *An. gambiae* heads under LD conditions from reanalyzed Rund *et al*. 2011 data [30] with a JTK_CYCLE q < 0.05 cutoff are compared to *Ae. aegypti* heads under LD conditions from Ptitsyn *et al*. 2011 [34] data also reanalyzed with JTK_CYCLE q < 0.05 cutoff. For each biological functional category, the number of genes found rhythmic in *An. gambiae*, the number of those genes where a homologue was identified in *Ae. aegypti,* and finally the number of those homologues that were found rhythmic in *Ae. aegypti* is provided.

We then looked at individual categories of genes to compare their expression patterns between *Aedes and Anopheles,* and report here on some of the categories of rhythmic genes that we found that had interesting differences or similarities in expression patterns between the two species. We hypothesize how differences in diel expression between the two species could be explained by differences in known circadian biology between the two species as has been suggested in other studies between animals in different temporal niches [24,105-108]. However, we acknowledge that as we are only comparing two species, this present analysis can only conclusively show the presence of a difference between the two species, and not the reason for such differences.

#### Temporal similarities and differences in V-ATPase gene expression between *An.* gambiae and *Ae. aegypti*

The multi-subunit vesicular-type ATPase (V-ATPase) that utilizes ATP to actively transport H^+^, has been detected in *Ae. aegypti* in the osmoregulatory tissues, including stomach, malpighian tubules, anterior hindgut and rectum [109]; in *An. funestus* salivary glands [110]; and in the antennal sensilla of the saturniid moth *Antheraea pernyi*[111]. Importantly, there is evidence for a role of V-ATPase in *Plasmodium* infection in *Aedes* and *Anopheles*, and dengue and Japanese encephalitis infections in *Aedes*[112-114]. We have previously commented on the relevance of coordinated rhythms in V-ATPase subunit expression specifically in the bodies of *An. gambiae*, and its potential relationship to *Plasmodium* infection of the mosquito mid-gut [30,114]. V-ATPase is also thought to play a crucial role in the function of synaptic vesicles, and indeed *Drosophila* mutant for the V_0_ subunit a1 have impaired neurotransmitter release [115-117]. This mutation also impacts the endolysosomal degradation mechanism in *Drosophila* eye photoreceptors [118], and the *Drosophila* B-subunit V-ATPase is rhythmic at the protein level in the eye [119]. In the head under LD conditions, and using DAVID [103,104] to identify orthologs, we found that 7 genes encoding 7 of the 12 subunits in *Anopheles* to be rhythmic and in phase, with all peaking in the late day/dusk. In *Aedes,* 12 of the subunit genes (that represent 10 of the 12 subunits) are rhythmic and also expressed at the same phase, but the peak in expression is in opposite phase to *An. gambiae*, occurring around dawn (Figure 4B). As V-ATPase subunit gene expression is rhythmic, our analysis highlights the possibility that susceptibility by *Aedes* to dengue and Japanese encephalitis viruses may vary by time of day. Furthermore, if the rhythms are in similar phase in *Aedes* bodies as they are in their heads, it is likely that these mosquitoes up-regulate their V-ATPase at times when significant osmotic changes induced by a blood or sugar meal at differing if not opposite times of the day from *Anopheles* may occur, concordant with known differences in behavioral rhythms. Finally, as V-ATPase plays an important role in synaptic activity, it is possible that neuronal activity is modulated in a time-of-day manner in the two species, yet in opposite phases, again concordant with the differing times of behavioral activity in each mosquito.

#### Temporal similarities in vision gene expression between *An. gambiae* and *Ae. aegypti*

**Figure 5:**
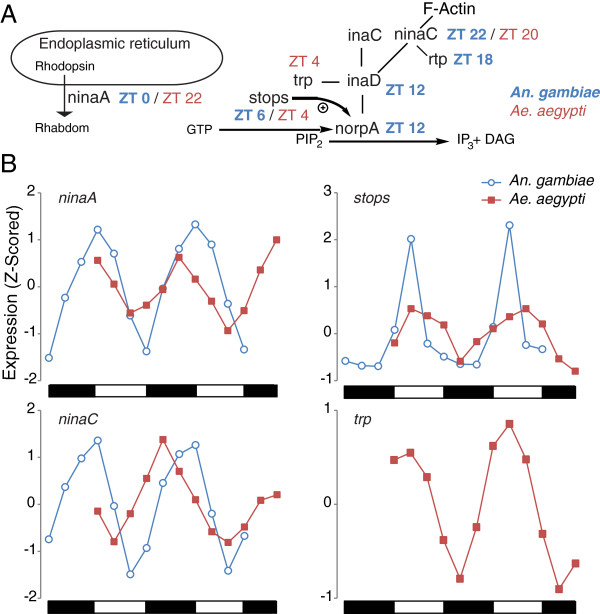
**Multiple components of the visual transduction cascade are rhythmically expressed and in similar phases in both *An. gambiae *and *Ae. aegypti. ***(**A**) Mosquito homologues to genes in the *Drosophila* phototransduction cascade signaling complex were identified as rhythmic in LD heads using the JTK_CYCLE algorithm (q < 0.05). Peak phase in expression is indicated next to gene names as Zeitgeber time (ZT) with *An. gambiae* genes in bold blue and *Ae. aegypti* genes in red. (**B**) Transcription profiles of genes rhythmic in both species as well as *Ae. aegypti transient receptor potential* (*trp*)*. An. gambiae trp* expression was not detected above background levels. Expression values are Z-scored. Day and night are indicated by the horizontal white/black bars below the chart. The shift in the presentation of the beginning and end of expression profiles reflects differences in experimental design between *Anopheles* and *Aedes* collections. Mosquito visual gene identities and functions are based on homology to *Drosophila* and are presumed similar in *Anopheles* and *Aedes* mosquitoes [120,121,126]*.* For the full set of *An. gambiae* vision genes found rhythmic, see Rund *et al.* 2011 [30]. All data shown are from LD heads.

We next looked at genes involved in the visual transduction pathway, using the *Drosophila* visual signaling pathway [120-122] as a model to identify mosquito orthologs, and identify genes rhythmic in both *Anopheles* and *Aedes* (Figure 5). The eye specific *ninaA/cyclophilin-r* (AGAP009991/AAEL009421) encoding an eye-specific cyclophilin which is involved in rhodopsin transport from the endoplasmic reticulum [123], peaks in expression in both *Anopheles* and *Aedes* in the early morning phase. Particularly interesting is the inaD signaling complex. The inaD protein organizes components of the phototransduction cascade into a signaling complex that contains, among other components, the kinase/myosin hybrid, ninaC (AGAP009730/AAEL000596). Expression of *ninaC* is rhythmic in both species, peaking at mid- to late night. In *Anopheles,* but not *Aedes*, expression of *inaD* (AGAP002145/AAEL008705) itself, as well as another gene encoding a component of the signaling complex, *retinophilin* (*rtp,* AGAP003547/AAEL000457) is rhythmic [30]*.* In *Aedes* however, the major light-gated ion channel, *trp* (AAEL005437), is rhythmic, peaking in expression in the early morning. Expression of *trp* (AGAP000348) was not detected on our *An. gambiae* microarray. Finally, in both *Anopheles* and *Aedes, stops* (AGAP000213/AAEL005443) is rhythmically expressed, peaking at mid-day. The PLCβ regulator, STOPS, is critical for maintaining protein, but not mRNA, levels of NORPA [124] suggesting conserved rhythmic control of visual signal transduction could be tightly regulated by NORPA through rhythmic expression of *norpA* as well as through STOPS*.* The rhythmic gene expression of visual transduction proteins in *Anopheles* and *Aedes* might contribute to a conserved time-of-day specific gating mechanism for tuning sensitivity to photic activation of the mosquito visual system irrespective of temporal niche (i.e. nocturnal versus diurnal) to match the daily changes in light levels. This is consistent with electrophysiological studies in numerous other insect species [125]. Organisms that fail to adjust their sensitivity to light in a time-of-day manner will have visual systems too insensitive during the night and overly sensitive during the day [125].

#### Temporal differences in aminoacyl-tRNA synthetases and olfaction gene expression between *An. gambiae* and *Ae. aegypti*

**Figure 6:**
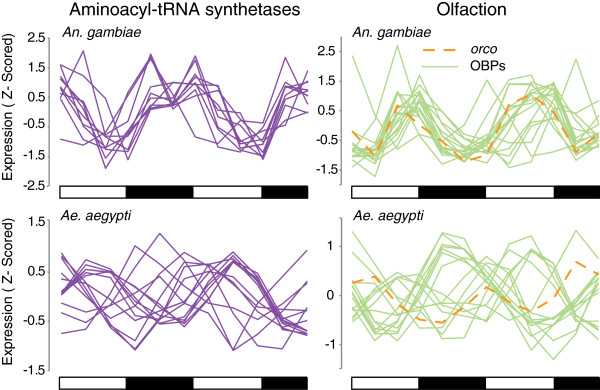
**Multiple aminoacyl-tRNA synthetases and olfaction genes are rhythmic in both *An. gambiae *and *Ae. aegypti.*** Expression profiles of all aminoacyl-tRNA synthetases and OBPs that were detected as rhythmic (q < 0.05), and *orco* (q = 0.06). *An. gambiae* appears to have tighter co-regulation of gene expression than *Ae. aegypti*. Expression data have been Z-scored. Aminoacyl-tRNA synthetases predicted using DAVID [103,104], *Ae. aegypti* OBPs from Zhou *et al.*[127], and *An. gambiae* OBPs are annotated in VectorBase. All data from LD heads. As *Anopheles* collection began at dusk (ZT 12) and *Ae. aegypti* collection at dawn (ZT 0), the second and third timepoints from the *Anopheles* collection are appended to the end of the collection as the last two timepoints for visualization purposes. Day and night are indicated by the horizontal white/black bars below the charts. All data shown are from LD heads.

We next compared gene expression between the putative aminoacyl-tRNA synthetases that “prime” amino acids to tRNA, and the olfaction genes *OBP*s and *odorant receptor coreceptor* (*orco*), between the two mosquito species. In *An. gambiae*, we found significant rhythmic co-regulation with 11 rhythmic aminoacyl-tRNA synthetases (q < 0.05) that all peak approximately in phase toward the later part of the night in LD heads (Figure 6). In *An. aegypti*, we find 15 aminoacyl-tRNA synthetases are rhythmic (Figure 6), and that there is an enrichment in genes peaking in expression towards the middle of the day (antiphasic to *Anopheles*) but with several exceptions (genes peaking in expression at other times of the day). Observed rhythms in aminoacyl-tRNA synthetases would suggest that mosquitoes have increased protein synthesis activity during their behavioral inactive periods. This could correspond with the rebuilding of cellular products while the mosquito rests, as well as anticipation of large amounts of protein synthesis involved in egg development that follow a blood meal. Our results indicate there may be rhythmic control at the translational level which produces, enhances or modifies 24 hr rhythms downstream of gene expression. As the peak in expression of the aminoacyl-tRNA synthetases is different between the nocturnal *An. gambiae* and diurnal *Ae. aegypti,* we hypothesize that in both species, expression of aminoacyl-tRNA synthetases is upregulated prior to the mosquitoes’ inactive phase in preparation for increases in protein synthesis while the mosquito is in a rest state.

A similar pattern of co-regulation existed in the olfactory genes that we examined. In *An. gambiae* there appears to be very tight regulation among the 17 rhythmic *OBP*s (q < 0.05), with a majority peaking in expression around dusk. However, the 15 rhythmic *OBP*s in *Ae. aegypti* peak in expression at various times of the day, instead of clustering around a particular phase of the LD cycle. This finding may be related to when there may be temporal segregation of behaviors requiring the detection of discrete odors. Finally, we compared the expression of the gene encoding the master olfactory heterodimer required for all odorant receptor transduction, *odorant receptor coreceptor* (*orco*) between the two species (AGAP002560/AAEL005776) [128]. Note in *An. gambiae*, *orco* is also known as *odorant receptor 7* (*OR7*). We find that *orco* (q = 0.06) peaks in *An. gambiae* at ZT 10, which is immediately prior to dusk (ZT 12) and the onset of nocturnal behavioral activities involving olfaction, *i.e.* host seeking, blood feeding, nectar feeding and oviposition [3-12]. However, *orco* peaks in the morning at ZT 3 in *Ae. aegypti*, which may be consistent with this species being most active during the day time [15,16,21,25,101].

## Conclusions

Mosquitoes exhibit 24 hr time-of-day specific rhythms in flight activity, feeding and reproductive behaviors and developmental processes. To understand the molecular basis for these rhythms in *An. gambiae*, we have utilized microarray analysis on 48 hr time courses collected from female heads and bodies. Recent studies have highlighted a broad diversity of 24 hr rhythmic gene expression in nocturnal *An. gambiae* and diurnal *Ae. aegypti* mosquitoes, although no previous comparison of rhythmic genome-wide expression between the two temporally segregated species has been made. In *An. gambiae*, many genes are rhythmic only in an environmental LD cycle suggesting direct regulation of gene expression by light, whilst others are rhythmic under DD conditions, revealing regulation by the endogenous circadian clock. In time courses from *An. gambiae* head and body under LD and DD conditions, we applied three algorithms that detect sinusoidal patterns and an algorithm that detects spikes in expression. This revealed across four experimental conditions 393 probes newly scored as rhythmic. These genes correspond to functions such as metabolic detoxification, immunity and nutrient sensing. Included are *GSTE5*, whose expression pattern and chromosomal location are shared other with other GSTs, and suggests shared chromosomal regulation; the pulsatile expression of *CYP6M2*, a cytochrome P450 that metabolizes pyrethroid insecticides; and the *Anopheles* homologue to *Drosophila sugarbabe*, a regulator of insulin synthesis. Time course expression profiles and cosine wave-fitting algorithm data for all probes can be viewed on our publically accessible database, *Bioclock*[58]. In total, between the present study and our previous we have revealed under LD conditions, 1424 and 726 rhythmic genes with a period length of 20–28 hr in the head and body, respectively; and under DD conditions, 928 rhythmic genes in the head and 510 in the body with an 18.5-26 hr period length.

We explored the interaction of light and the circadian clock and highlight the regulation of OBPs that are important components of the olfactory system. We reveal that *OBP*s have unique expression patterns as mosquitoes make the transition from LD to DD conditions, and propose a model for the three distinct patterns of expression that we observe. Finally, we compared rhythmic expression between time courses of *An. gambiae* and *Ae. aegypti* heads collected under LD conditions using a single cosine fitting algorithm, and report distinct similarities and differences in the temporal regulation of genes involved in key processes such as protein synthesis (specifically tRNA priming), the V-ATPase and in the sensory modalities of olfaction and vision. We propose that the similarities and differences shared between the two species may in part reflect their distinct temporal niches, although they also have differences in habitat and evolutionary lineages which likewise could be underlying the differences we report [52,53].

These data build on our previous analyses of the time-of-day specific regulation of the *An. gambiae* transcriptome. Improved understanding of the molecular basis for circadian- and light-regulated rhythms that underlie key physiological aspects of mosquito vectors may prove to be important to successful implementation of established and novel vector control methods. Rhythmic changes in genes associated with susceptibilities to immune and insecticidal challenges, sensory physiology and feeding behavior may provide opportunities for new control strategies, including gene manipulation by generation of transgenic mosquitoes [129,130]. Other important implications of such extensive rhythmic regulation includes the efficacy of sterile insect technique/pathogen-resistant strains, where differences in diel timing of mating between reared and wild populations would limit their success [129-132]. Moreover, the use of insecticide impregnated bed nets may be acting as a selective pressure that is modifying the age/genetic composition of the population and the time when nocturnal anopheline vectors initiate host-seeking behavior such that it occurs at a different time of the night [59,133]. These considerations illustrate the need for a better understanding of the circadian biology of these disease vector species.

## Methods

### Microarray gene expression data

*An. gambiae* microarray data collection and analysis were originally reported in Rund *et al.* 2011 [30]. In that study, female mated, but not blood-fed, Pimperena S molecular form mosquitoes were collected every 4 hr over 48 hr under either LD or DD conditions, heads separated from bodies, RNA extracted, and RNA expression levels determined using the Affymetrix platform (*Plasmodium*/*Anopheles* Affymetrix GeneChips, Affymetrix 900511). Expression data is deposited in GEO Express (accession no. GSE22585), VectorBase Expression Data BioMart [134] and are graphically available and easily searchable at our website, *Bioclock*[58].

*Ae. aegypti* microarray data collection and analysis was originally reported in Ptitsyn *et al.* 2011 [34]. In this study, female mosquitoes (as a mixed population from 11 separate F_5_ colonies originally derived from wild caught populations from Chiapas, Mexico) were collected in duplicate every 4 hr over 24 hr under LD conditions, RNA extracted from heads, and RNA expression levels determined using Agilent microarrays described in Xi *et al.*[135]. Normalized gene expression data as 44 hr profiles was available as supplementary material to their publication [34], and reanalyzed and presented here as a derivative work in accordance with the terms of the Creative Commons 2.0 license and the BioMedical Central open access charter.

### Application of COSOPT, JTK_CYCLE and discrete Fourier transform algorithms for generating a consensus rhythmic gene list

*An. gambiae* microarray data comprised two replicate samples. Only *An. gambiae* expression profiles where mean fluorescent intensity > 20 (which would exclude 99% of *Plasmodium falciparum* probes contained on the microarray) in both replicates were considered. No fluorescent intensity cutoff was applied to *Ae. aegypti* probes.

Software implementations of the COSOPT [30,35-38,42,43] and JTK_CYCLE [44-46] algorithms were used as previously described. Briefly, COSOPT measures the goodness-of-fit between experimental data and a series of cosine curves with varying phases and (user defined) period lengths. Multiple means corrected β (pMMCβ) values are determined by scrambling experimental data and re-fitting it to cosine curves to determine probability that the observed data matches a cosine curve by chance alone. JTK_CYCLE is a nonparametric statistical algorithm designed to identify and characterize cycling variables in large data sets. It applies the Jonckheere-Terpstra-Kendall (JT) test and Kendall’s tau (rank correlation), finding the optimal combination of period and phase that minimizes the p-value of Kendall tau correction between the experimental time series and each tested cyclical ordering, this being derived from cosine curves. JTK_CYCLE generates period length and phase estimates, as well as corrects for multiple comparisons *post hoc* (the p-value for a given probe is converted to a more stringent Benjamini-Hochberg q-value, which takes into consideration the possible false positive rate across all probes.) A measure of rhythm amplitude is also determined and reflects the 1-cycle median sign-adjusted deviation from the median in relation to the optimal cosine pattern [for a perfect cosine wave, this is amplitude/sqrt(2), where amplitude is defined as the median absolute deviation from the median].

For COSOPT and JTK_CYCLE, only probes with an average computed period length of between 18.5 - 26.5 hr for constant condition experiments or 20-28 hr for LD experiments in both replicate time courses were considered. The multiple means corrected β (pMMCβ) value, p, was used as the described COSOPT cutoff value. In this paper, described p value cutoffs are the averages of the two COSOPT-generated p values from each of the replicate time courses.

For JTK _CYCLE, the Benjamini-Hochberg q-value was used as the described JTK_CYCLE cutoff value. JTK_CYCLE accounts for replicate samples, thus only one q value is generated.

For discrete Fourier transform (DFT), the relative amplitude of the 24 hr period frequency component was calculated with the discrete Fourier transform [136]. First the time series data was transformed by

$$X=\left|\mathrm{DFT}\left(x\right)\right|$$

where x is the time series signal and X is a vector of the sinusoidal amplitudes. To mitigate the effects of the mean fluorescent intensity, X[0] was set to zero. Note that since the sampling rate is 4 hr with a window of 48 hr, X has seven tuples and each value defines the amplitude of an N/48hr embedded frequency where N is the index. Thus, as period lengths deviate farther from 24 hr, they are less likely to be discovered by this method. This becomes particularly apparent under DD conditions.

The relative amplitude of the 24 hr period (1/24 hr frequency) component characterized the presence of that sinusoid in the data. This was calculated by

$$s=X\left[2\right]/\left|X\right|$$

ensuring that the value would range between zero and one. For any described s value cutoff, the average of the s values returned from the two replicate time courses is considered.

### Pattern matching to search for pulsatile expression patterns

Pulsatile patterns were discovered by convolving a template with the expression signals [137]. The template, which corresponds to spikes in expression, 24 hr apart, was defined mathematically as

$$T=\left[1.0\;-0.4\;-0.4\;-0.4\;-0.4\;-0.4-\;1.0\right].$$

These values were chosen such that convolution with unity (constitutive, non-cyclic expression) is 0 and the peak samples are weighted more than the valleys. Prior to convolution, the signals were gamut normalized then reduced by the mean value of the signal. Convolution yielded a c value for each of the 13 time points; the maximum c value was used to represent the maximum pulsatile expression for each given expression pattern across the 13 time points. Expression profiles were considered pulsatile where c > 1.6 and where peak-to-trough fold change > 1.5 in both replicates. The c value cutoff was determined through manual inspection as the threshold at which no apparent false-positives were detected. Note c has a magnitude and a sign. High-magnitude, positive values reflect a good match to the template whereas small magnitude values reflect a poor match to the template.

### Gene annotation

Where possible, we used the *An. gambiae* identifications from our previous report [30]. For genes not previously annotated, we used the same naming conventions. Briefly, genes were annotated primarily from information stored at VectorBase, often using the closest homologue from *Ae. aegypti* (AAEG:)*, Cx. quinquefasciatus* (QQUI:)*, D. melanogaster* (DMEL:) or *Caenorhabditis elegans* (CELG:) (in that order), but also using published literature and the Database for Annotation, Visualization and Integrated Discovery (DAVID) to match putative *An. gambiae* genes to enzymatic pathways [103,104,134]. Where no *An. gambiae* or orthologous gene name was available, InterProScan [138] was used to annotate genes; a representative InterPro or the associated Gene Ontology (GO) term may be provided. *Ae. aegypti* gene names were identified in a similar manner. *Ae. aegypti* OBPs were identified from Zhou *et al*. 2008 [127]. Gene annotations correspond with the July 3, 2012 VectorBase release. Genes that have been previously annotated by others in *An. gambiae,* but not in VectorBase, appear in the text with an ‘*ag*’ prefix.

### Hierarchical cluster analysis

Hierarchical cluster analysis was performed using Cluster 3.0 and visualized using Java TreeView [139,140]. Data were log_2_ transformed, mean centered and normalized across the time course for each gene and clustered (centroid linkage). For *An. gambiae,* only probes that had a mean fluorescence intensity across all 13 timepoints >20 were analyzed.

### Real-time quantitative RT-PCR (qRT-PCR) analysis

Total RNA was treated with DNaseI (Qiagen, Valencia, CA) and used for cDNA synthesis using a High Capacity cDNA reverse transcriptase kit (Applied Biosystems, Foster City, CA) primed with random hexamers. PCR thermocycling and qRT-PCR were performed as previously described [35] using SYBR green reagents with an Applied Biosystems 7500 Fast Real-Time PCR System and quantification based on the generation of standard curves or the delta-delta CT method. Dissociation curves to test for primer dimers were generated using dissociation curve software (Applied Biosystems). Normalization of genes was calculated relative to *ribosomal protein S7* (*RPS7*). Real-time quantitative RT-PCR Primer sequences (5’ → 3’): *RPS7* (AGAP010592) F: CATTCTGCCCAAACCGATG, R: AACGCGGTCTCTTCTGCTTG, from Dana *et al*. 2005 [141]; *CYP6M2* (AGAP008212) F: GTATGATGCAGGCCCGTATAG R: GCCATAATGAAACTCTCCTTCG from Müller *et al.* 2007 [142]; *OBP3* (AGAP001409) F: GATTCGTGCTGGAGCTCGAG, R: GTAAAAAGTAGTGCACCGGGTCC; *OBP6* (AGAP003530) F: CATGCTTAATGGATCTAACACAAAC, R: GCGACTTCACAGCGATCC from Biessmann *et al.* 2005 [143].

### Promoter search analysis

A search for defined consensus sequences [49,88-94] was performed at the University of Notre Dame Genomics and Bioinformatics Core Facility using a custom written Perl script on the upstream regions of type I OBPs, type II OBPs and the other genes found clustering with those OBPs (see Figure 3) and type III OBPs. In general, we utilized the promoter element search criteria of Claridge-Chang *et al*. [49]. The upstream region of each gene of interest (up to 5kb unless there was overlap with the predicted coding region of another gene) was downloaded from VectorBase [134]. See Additional file 6 for specific consensus sequences and search criteria.

### Comparison of genes rhythmic in *An. gambiae* versus *Ae. aegypti*

In order to make as similar as possible comparison of rhythmic gene expression between the two species, from experiments of slightly different design, we reanalyzed both datasets using JTK_CYCLE with a stringent q < 0.05 probability cutoff of genes with a 20-28 hr period. Using the list of gene homology maintained at VectorBase, homologues to all rhythmic *An. gambiae* genes were identified in *Ae. aegypti.* Homologues were then compared against the rhythmic *Ae. aegypti gene* list and matches noted. For both *An. gambiae* and *Ae. aegypti* the probe with the lowest q value was considered. The *Ae. aegypti* homologues considered were the homologues listed in VectorBase with the highest percent identity that were rhythmic (q < 0.05).

## Abbreviations

CB: Clock box; CCG: Clock controlled gene; DD: Constant dark; CRE: Ca^2+^/cAMP response element; DFT: Discrete Fourier transform; GST: Glutathione S-transferase; LB: Light box; LD: Light:dark cycle; OBP: odorant binding protein; TTFL: Transcriptional - translational feedback loop; ZT: Zeitgeber time.

## Competing interests

The authors declare no competing interests.

## Authors’ contributions

SSCR performed *Anopheles* and *Aedes* gene expression analysis, hierarchical cluster analysis, qRT-PCR and drafted the manuscript. JEG implemented the pattern matching algorithm, discrete Fourier transform and compared *Anopheles* and *Aedes* expression. GED conceived of the study and participated in its design, coordination and analysis and co-wrote the manuscript. All authors read and approved the final manuscript.

## Supplementary Material

Additional file 1 **Rhythmic *An. gambiae *probes, by statistical test cutoff value.** Only probes with a mean fluorescent intensity >20 across the time course were analyzed. Probes indicated as rhythmic using COSOPT or DFT were found rhythmic in both of the two replicate runs. In JTK_CYCLE and COSOPT, only probes where period length under LD conditions was between 20 hr to 28 hr or in DD conditions between 18.5 hr - 26.5 hr are reported. Note DFT performed on 24 hr signal for all runs, see methods for more details. Click here for file

Additional file 2 **Analysis of expression data by various algorithms reveals overlap in *An. gambiae *probes deemed rhythmic.** Venn diagrams show the number of probes in LD bodies and DD heads and bodies identified as rhythmic using the JTK_CYCLE, DFT and COSOPT algorithms at the statistical cutoffs indicated. In LD bodies, a total of 808 probes were identified as rhythmic using all three algorithms, representing 148 new rhythmic probes from those identified previously [30]. In DD heads, a total of 517 probes were found rhythmic using all three conditions (47 new probes). In DD bodies, a total of 332 probes were identified as rhythmic using all three algorithms (32 new probes). Note DFT analysis limits the number of probes that may be deemed rhythmic under DD conditions; see methods for more information. See Figure 1 for LD head Venn diagram. See Additional file 3 for list of probes newly identified as rhythmic. The numbers outside the Venn diagrams represent the number of probes with a mean fluorescent intensity above background that were not scored as rhythmic by any of the algorithms. Click here for file

Additional file 3 ***An. gambiae *probes found rhythmic by COSOPT, JTK_CYCLE and DFT but not in the original COSOPT analysis.** List of probe identities for LD heads, DD heads, LD bodies and DD bodies found rhythmic with pMMCβ < 0.2 (COSOPT), q < 0.1 (JTK_CYCLE), and s > 0.3 (DFT), but that were not found rhythmic using the original COSOPT statistical cutoff of pMMCβ < 0.1 [30]. Only probes where the mean fluorescent intensity was >20 across all timepoints were considered. Annotation, probe ID, COSOPT pMMCβ and peak phase (ZT), JTK_CYCLE q and peak phase (ZT) and DFT s values are provided. Probes that do not map to current genes are marked as “unassociated.” Probes that map to more than one gene are marked with an asterisk. Click here for file

Additional file 4 ***An. gambiae *gene expression changes in LD versus DD conditions.** (A) qRT-PCR confirmation of reduction in expression in *OBP3* and *OBP6* under DD versus LD conditions. Values are mean ± SD of gene expression as a percentage normalized to the LD value of 100%. Female mated, non-blood fed mosquitoes, 5-7 day post emergence from mosquitoes reared concurrently under different lighting conditions were collected under LD conditions or DD conditions (from mosquitoes placed in darkness for 24 hr) at ZT/CT 12. (B) Average gene expression changes between LD and DD conditions as measured by microarray analysis across 44 hr. The average expression level averaged across all 12 time points was analyzed in both LD and DD, and the fold change difference in expression level between LD and DD determined. Most probes showed similar expression levels between LD and DD. However, significant variation occured in a subset of genes. This was especially pronounced in bodies, where 47% of the rhythmic genes had >2 fold difference in expression levels between LD and DD conditions. Click here for file

Additional file 5 **Amplitude measures for *An. gambiae *clock genes expressed in the head under LD and DD conditions.** Amplitudes calculated as peak divided by nadir normalized fluorescence values and where peak-to-nadir occurred with an interval of 8-16 hr. The JTK_CYCLE amplitude value reflects the 1-cycle median sign-adjusted deviation from the median in relation to the optimal cosine pattern. (DOCX 16 kb) Click here for file

Additional file 6 **Promoter sequence search of light- and circadian- driven gene expression.** Specific promoter search criteria and the results of searching for defined response elements [49,88-95] in the 5kb 5' region upstream of the transcription start site of type I OBPs, type II OBPs and the other genes found clustering with those OBPs (see Figure 3), and type III OBPs. The table provides the gene name, VectorBase ID and the number and identity of consensus sequences found in the 1kb and 5kb upstream region of the genes. For some genes, the full 5kb region was not available, as it would overlap with the predicted coding region of another gene. In such cases, only the region that did not overlap was considered; the number of base pairs considered is provided in the “Upstream region (bp)” column. Click here for file

Additional file 7 **Rhythmic genes in heads under LD conditions that are common to both *An. gambiae *and *Ae. aegypti.*** 539 genes were identified as rhythmic (q < 0.05) in both *An. gambiae* and *Ae. aegypti*. For each pair of homologous rhythmic genes, an *An. gambiae* annotation, JTK_CYCLE phases and q values, probe IDs and gene IDs are provided. For both *An. gambiae* and *Ae. aegypti* the probe with the lowest q value is provided. The *Ae. aegypti* homologues to *An. gambiae* that are provided in the table are those listed in VectorBase with the highest percent identity, *that were also found rhythmic*. Click here for file

## References

[B1] Dunlap JC Loros JJ Decoursey PJ Chronobiology: Biological timekeeping 2004 Sunderland Mass: Sinauer Associates

[B2] Charlwood JD The swarming and mating behaviour of *Anopheles gambiae* s.s. (Diptera: Culicidae) from São Tomé Island J Vector Ecol 2002 27 178 183 12546454

[B3] Gary RE Jr Foster WA Diel timing and frequency of sugar feeding in the mosquito *Anopheles gambiae*, depending on sex, gonotrophic state and resource availability Med Vet Entomol 2006 20 308 316 10.1111/j.1365-2915.2006.00638.x 17044882

[B4] Jones MDR Gubbins SJ Changes in circadian flight activity of mosquito *Anopheles gambiae* in relation to insemination, feeding and oviposition Physiol Entomol 1978 3 213 220 10.1111/j.1365-3032.1978.tb00151.x

[B5] Reiter P Jones MDR Eclosion timing mechanism in the mosquito *Anopheles gambiae* J Entomol Ser A 1976 50 161 168

[B6] Jones MDR Reiter P Entrainment of pupation and adult activity rhythms during development in mosquito *Anopheles gambiae* Nature 1975 254 242 244 10.1038/254242a0 1113887

[B7] Jones MD Delayed effect of light on the mosquito "clock" Nature 1973 245 384 385 10.1038/245384a0 4593497

[B8] Jones MDR Cubbin CM Marsh D The circadian rhythm of flight activity of the mosquito *Anopheles gambiae*: The light-response rhythm J Exp Biol 1972 57 337 346

[B9] Jones MDR Hill M Hope AM The circadian flight activity of the mosquito *Anopheles gambiae*: Phase setting by the light regime J Exp Biol 1967 47 503 511 5592417 10.1242/jeb.47.3.503

[B10] Das S Dimopoulos G Molecular analysis of photic inhibition of blood-feeding in *Anopheles gambiae* BMC Physiol 2008 8 23 10.1186/1472-6793-8-23 19087335 PMC2646746

[B11] Fritz ML Ovipositional periodicity of caged *Anopheles gambiae* individuals J Circadian Rhythms 2008 6 2 10.1186/1740-3391-6-2 18221544 PMC2253508

[B12] Sumba LA Daily oviposition patterns of the African malaria mosquito *Anopheles gambiae* Giles (Diptera: Culicidae) on different types of aqueous substrates J Circadian Rhythms 2004 2 6 10.1186/1740-3391-2-6 15596009 PMC544589

[B13] Rund SSC Lee SJ Bush BR Duffield GE Strain- and sex-specific differences in daily flight activity and the circadian clock of *Anopheles gambiae* mosquitoes J Insect Physiol 2012 58 1609 1619 10.1016/j.jinsphys.2012.09.016 23068991

[B14] Keating JA Bhattacharya D Rund SSC Hoover S Dasgupta R Lee SJ Duffield GE Striker R Mosquito protein kinase G phosphorylates flavivirus NS5 and alters flight behavior in *Aedes aegypti* and *Anopheles gambiae* Vector Borne Zoonotic Dis 2013 13 in press 10.1089/vbz.2012.1110 PMC3741427 23930976

[B15] Corbet PS Smith SM Diel periodicities of landing of nulliparous and parous *Aedes aegypti* (L.) at Dar es Salaam, Tanzania (Diptera, Culicidae) Bull Entomol Res 1974 64 111 121 10.1017/S0007485300027036

[B16] Jones MDR The programming of circadian flight-activity in relation to mating and the gonotrophic cycle in the mosquito, *Aedes aegypti* Physiol Entomol 1981 6 307 313 10.1111/j.1365-3032.1981.tb00275.x

[B17] McClelland GAH Field observations on periodicity and site preference in oviposition by *Aedes aegypti* (L.) and related mosquitoes (Diptera: Culicidae) in Kenya Proc R Entomol Soc Lond Ser A Gen Entomol 1968 43 147 154

[B18] Tuchinda P Kitaoka M Ogata T Kurihara T On the diurnal rhythmus of biting behavior of *Aëdes aegypti* in relation to the age and to the hemorrhagic fever in Bangkok, 1964 Japan J Trop Med 1969 10 1 6

[B19] Harrington LC Ponlawat A Edman JD Scott TW Vermeylen F Influence of container size, location, and time of day on oviposition patterns of the dengue vector, *Aedes aegypti*, in Thailand Vector Borne Zoonotic Dis 2008 8 415 424 10.1089/vbz.2007.0203 18279006 PMC2978047

[B20] Kawada H Takagi M Photoelectric sensing device for recording mosquito host-seeking behavior in the laboratory J Med Entomol 2004 41 873 881 10.1603/0022-2585-41.5.873 15535615

[B21] Yee WL Foster WA Diel sugar-feeding and host-seeking rhythms in mosquitoes (Diptera: Culicidae) under laboratory conditions J Med Entomol 1992 29 784 791 1357175 10.1093/jmedent/29.5.784

[B22] Lardeux F Integrated control of peridomestic larval habitats of *Aedes* and *Culex* mosquitoes (Diptera: Culicidae) in atoll villages of French Polynesia J Med Entomol 2002 39 493 498 10.1603/0022-2585-39.3.493 12061446

[B23] Canyon DV Hii JLK Muller R Effect of diet on biting, oviposition, and survival of *Aedes aegypti* (Diptera: Culicidae) J Med Entomol 1999 36 301 308 10337099 10.1093/jmedent/36.3.301

[B24] Gentile C Rivas GBS Meireles-Filho ACA Lima JBP Peixoto AA Circadian expression of clock genes in two mosquito disease vectors: *cry2* is different J Biol Rhythms 2009 24 444 451 10.1177/0748730409349169 19926804

[B25] Chadee DD The diel oviposition periodicity of *Aedes aegypti* (L.) (Diptera: Culicidae) in Trinidad, West Indies: Effects of forced egg retention Bull Entomol Res 2010 100 599 603 10.1017/S0007485309990666 20178673

[B26] Naucke TJ Field evaluation of the efficacy of proprietary repellent formulations with IR3535 and picaridin against *Aedes aegypti* Parasitol Res 2007 101 169 177 10.1007/s00436-006-0450-2 17252270

[B27] Wong J Astete H Morrison AC Scott TW Sampling considerations for designing *Aedes aegypti* (Diptera:Culicidae) oviposition studies in Iquitos, Peru: Substrate preference, diurnal periodicity, and gonotrophic cycle length J Med Entomol 2011 48 45 52 10.1603/ME10149 21337947 PMC3108245

[B28] Gentile C Cloning and daily expression of the *timeless* gene in *Aedes aegypti* (Diptera: Culicidae) Insect Biochem Mol Biol 2006 36 878 884 10.1016/j.ibmb.2006.08.008 17046601

[B29] Chahad-Ehlers S Gentile C Lima JBP Peixoto AA Bruno RV Analysis of *cycle* gene expression in *Aedes aegypti* brains by *in situ* hybridization PLoS One 2013 8 e52559 10.1371/journal.pone.0052559 23300979 PMC3534671

[B30] Rund SSC Hou TY Ward SM Collins FH Duffield GE Genome-wide profiling of diel and circadian gene expression in the malaria vector *Anopheles gambiae* Proc Natl Acad Sci USA 2011 108 E421 E430 10.1073/pnas.1100584108 21715657 PMC3156198

[B31] Yuan Q Metterville D Briscoe AD Reppert SM Insect cryptochromes: Gene duplication and loss define diverse ways to construct insect circadian clocks Mol Biol Evol 2007 24 948 955 10.1093/molbev/msm011 17244599

[B32] Zhu H Yuan Q Froy O Casselman A Reppert SM The two CRYs of the butterfly Curr Biol 2005 15 R953 R954 10.1016/j.cub.2005.11.030 16332522

[B33] Mathias D Jacky L Bradshaw WE Holzapfel CM Geographic and developmental variation in expression of the circadian rhythm gene, *timeless*, in the pitcher-plant mosquito, *Wyeomyia smithii* J Insect Physiol 2005 51 661 667 10.1016/j.jinsphys.2005.03.011 15979087

[B34] Ptitsyn A Rhythms and synchronization patterns in gene expression in the *Aedes aegypti* mosquito BMC Genomics 2011 12 153 10.1186/1471-2164-12-153 21414217 PMC3072344

[B35] Duffield GE Circadian programs of transcriptional activation, signaling, and protein turnover revealed by microarray analysis of mammalian cells Curr Biol 2002 12 551 557 10.1016/S0960-9822(02)00765-0 11937023

[B36] Panda S Coordinated transcription of key pathways in the mouse by the circadian clock Cell 2002 109 307 320 10.1016/S0092-8674(02)00722-5 12015981

[B37] Ceriani MF Genome-wide expression analysis in *Drosophila* reveals genes controlling circadian behavior J Neurosci 2002 22 9305 9319 12417656 10.1523/JNEUROSCI.22-21-09305.2002 PMC6758054

[B38] Straume M DNA microarray time series analysis: Automated statistical assessment of circadian rhythms in gene expression patterning Methods Enzymol 2004 383 149 166 15063650 10.1016/S0076-6879(04)83007-6

[B39] Khan S Rowe SC Harmon FG Coordination of the maize transcriptome by a conserved circadian clock BMC Plant Biol 2010 10 126 10.1186/1471-2229-10-126 20576144 PMC3095283

[B40] Hughes ME Harmonics of circadian gene transcription in mammals PLoS Genet 2009 5 e1000442 10.1371/journal.pgen.1000442 19343201 PMC2654964

[B41] Covington MF Maloof JN Straume M Kay SA Harmer SL Global transcriptome analysis reveals circadian regulation of key pathways in plant growth and development Genome Biol 2008 9 R130 10.1186/gb-2008-9-8-r130 18710561 PMC2575520

[B42] Sato TK A functional genomics strategy reveals Rora as a component of the mammalian circadian clock Neuron 2004 43 527 537 10.1016/j.neuron.2004.07.018 15312651

[B43] Stevenson BJ Cytochrome P450 6M2 from the malaria vector *Anopheles gambiae* metabolizes pyrethroids: Sequential metabolism of deltamethrin revealed Insect Biochem Mol Biol 2011 41 492 502 10.1016/j.ibmb.2011.02.003 21324359

[B44] Hughes ME Hogenesch JB Kornacker K JTK_CYCLE: An efficient nonparametric algorithm for detecting rhythmic components in genome-scale data sets J Biol Rhythms 2010 25 372 380 10.1177/0748730410379711 20876817 PMC3119870

[B45] Hughes ME Grant GR Paquin C Qian J Nitabach MN Deep sequencing the circadian and diurnal transcriptome of *Drosophila* brain Genome Res 2012 22 1266 1281 10.1101/gr.128876.111 22472103 PMC3396368

[B46] Xu K The circadian clock interacts with metabolic physiology to influence reproductive fitness Cell Metab 2011 13 639 654 10.1016/j.cmet.2011.05.001 21641546 PMC3152999

[B47] Keegan KP Pradhan S Wang JP Allada R Meta-analysis of *Drosophila* circadian microarray studies identifies a novel set of rhythmically expressed genes PLoS Comput Biol 2007 3 e208 10.1371/journal.pcbi.0030208 17983263 PMC2098839

[B48] Wijnen H Naef F Boothroyd C Claridge-Chang A Young MW Control of daily transcript oscillations in *Drosophila* by light and the circadian clock PLoS Genet 2006 2 e39 10.1371/journal.pgen.0020039 16565745 PMC1413497

[B49] Claridge-Chang A Circadian regulation of gene expression systems in the *Drosophila* head Neuron 2001 32 657 671 10.1016/S0896-6273(01)00515-3 11719206

[B50] Michael TP Network discovery pipeline elucidates conserved time-of-day-specific cis-regulatory modules PLoS Genet 2008 4 e14 10.1371/journal.pgen.0040014 18248097 PMC2222925

[B51] Lin Y Influence of the *period*-dependent circadian clock on diurnal, circadian, and aperiodic gene expression in *Drosophila melanogaster* Proc Natl Acad Sci USA 2002 99 9562 9567 10.1073/pnas.132269699 12089325 PMC123180

[B52] Harbach RE The Culicidae (Diptera): A review of taxonomy, classification and phylogeny Zootaxa 2007 1668 591 638

[B53] Clements AN The biology of mosquitoes 1999 Oxon: CABI Publ

[B54] Ranson H Pyrethroid resistance in African anopheline mosquitoes: What are the implications for malaria control? Trends Parasitol 2011 27 91 98 10.1016/j.pt.2010.08.004 20843745

[B55] The malERA Consultative Group on Drugs A research agenda for malaria eradication: Drugs PLoS Med 2011 8 e1000402 21311580 10.1371/journal.pmed.1000402 PMC3026688

[B56] Alonso PL A research agenda to underpin malaria eradication PLoS Med 2011 8 e1000406 10.1371/journal.pmed.1000406 21311579 PMC3026687

[B57] Miller BH Circadian and CLOCK-controlled regulation of the mouse transcriptome and cell proliferation Proc Natl Acad Sci USA 2007 104 3342 3347 10.1073/pnas.0611724104 17360649 PMC1802006

[B58] Rund SSC Duffield GE Bioclock Database of circadian gene expression. Developed by the Duffield laboratory 2011 Notre Dame, IN: University of Notre Dame http://nd.edu/~bioclock

[B59] Moiroux N Changes in *Anopheles funestus* biting behavior following universal coverage of long-lasting insecticidal nets in Benin J Infect Dis 2012 206 1622 1629 10.1093/infdis/jis565 22966127

[B60] Ortelli F Rossiter LC Vontas J Ranson H Hemingway J Heterologous expression of four glutathione transferase genes genetically linked to a major insecticide-resistance locus from the malaria vector *Anopheles gambiae* Biochem J 2003 373 957 963 10.1042/BJ20030169 12718742 PMC1223529

[B61] Ueda HR Genome-wide transcriptional orchestration of circadian rhythms in *Drosophila* J Biol Chem 2002 277 14048 14052 10.1074/jbc.C100765200 11854264

[B62] Lima-Camara TN Dengue infection increases the locomotor activity of *Aedes aegypti* females PLoS One 2011 6 e17690 10.1371/journal.pone.0017690 21408119 PMC3050906

[B63] Varghese J Lim SF Cohen SM *Drosophila* miR-14 regulates insulin production and metabolism through its target, *sugarbabe* Genes Dev 2010 24 2748 2753 10.1101/gad.1995910 21159815 PMC3003191

[B64] Puig O Marr MT Ruhf ML Tjian R Control of cell number by *Drosophila* FOXO: downstream and feedback regulation of the insulin receptor pathway Genes Dev 2003 17 2006 2020 10.1101/gad.1098703 12893776 PMC196255

[B65] Djouaka RF Expression of the cytochrome P450s, *CYP6P3* and *CYP6M2* are significantly elevated in multiple pyrethroid resistant populations of *Anopheles gambiae s.s*. from Southern Benin and Nigeria BMC Genomics 2008 9 538 10.1186/1471-2164-9-538 19014539 PMC2588609

[B66] Seay DJ Thummel CS The circadian clock, light, and cryptochrome regulate feeding and metabolism in *Drosophila* J Biol Rhythms 2011 26 497 506 10.1177/0748730411420080 22215608 PMC4537652

[B67] Kawada H Takemura SY Arikawa K Takagi M Comparative study on nocturnal behavior of *Aedes aegypti* and *Aedes albopictus* J Med Entomol 2005 42 312 318 10.1603/0022-2585(2005)042[0312:CSONBO]2.0.CO;2 15962780

[B68] Dunlap JC A circadian clock in *Neurospora*: How genes and proteins cooperate to produce a sustained, entrainable, and compensated biological oscillator with a period of about a day Cold Spring Harb Symp Quant Biol 2007 72 57 68 10.1101/sqb.2007.72.072 18522516 PMC3683860

[B69] Albers HE Liou SY Ferris CF Stopa EG Zoeller RT Klein DC, Moore RY, Reppert SM Neurochemistry of circadian timing Suprachiasmatic nucleus: The mind's clock 1991 New York: Oxford University Press 263 288

[B70] Inouye SIT Circadian rhythms of neuropeptides in the suprachiasmatic nucleus Progress in Brain Reserach 1996 111 75 90 10.1016/s0079-6123(08)60401-x 8990908

[B71] Chen CH Ringelberg CS Gross RH Dunlap JC Loros JJ Genome-wide analysis of light-inducible responses reveals hierarchical light signalling in *Neurospora* EMBO J 2009 28 1029 1042 10.1038/emboj.2009.54 19262566 PMC2683703

[B72] Hastings JW Biochemical aspects of rhythms - phase shifting by chemicals Cold Spring Harb Symp Quant Biol 1960 25 131 143 10.1101/SQB.1960.025.01.012 13712194

[B73] Biessmann H Nguyen QK Le D Walter MF Microarray-based survey of a subset of putative olfactory genes in the mosquito *Anopheles gambiae* Insect Mol Biol 2005 14 575 589 10.1111/j.1365-2583.2005.00590.x 16313558

[B74] Li ZX Pickett JA Field LM Zhou JJ Identification and expression of odorant-binding proteins of the malaria-carrying mosquitoes *Anopheles gambiae* and *Anopheles arabiensis* Arch Insect Biochem Physiol 2005 58 175 189 10.1002/arch.20047 15717318

[B75] Pelosi P Calvello M Ban L Diversity of odorant-binding proteins and chemosensory proteins in insects Chem Senses 2005 30 Suppl 1 i291 i292 15738163 10.1093/chemse/bjh229

[B76] Nichols Z Vogt RG The SNMP/CD36 gene family in Diptera, Hymenoptera and Coleoptera: *Drosophila melanogaster*, *D. pseudoobscura*, *Anopheles gambiae*, *Aedes aegypti*, *Apis mellifera*, and *Tribolium castaneum* Insect Biochem Mol Biol 2008 38 398 415 10.1016/j.ibmb.2007.11.003 18342246

[B77] Enayati A Ranson H Hemingway J Insect glutathione transferases and insecticide resistance Insect Mol Biol 2005 14 3 8 10.1111/j.1365-2583.2004.00529.x 15663770

[B78] Pace KE Baum LG Insect galectins: Roles in immunity and development Glycoconj J 2002 19 607 614 14758086 10.1023/B:GLYC.0000014092.86763.2f

[B79] Edery I Zwiebel LJ Dembinska ME Rosbash M Temporal phosphorylation of the *Drosophila* period protein Proc Natl Acad Sci USA 1994 91 2260 2264 10.1073/pnas.91.6.2260 8134384 PMC43350

[B80] Hardin PE Hall JC Rosbash M Feedback of the *Drosophila period* gene product on circadian cycling of its messenger RNA levels Nature 1990 343 536 540 10.1038/343536a0 2105471

[B81] Sehgal A Rhythmic expression of *timeless*: A basis for promoting circadian cycles in *period* gene autoregulation Science 1995 270 808 810 10.1126/science.270.5237.808 7481772

[B82] Rosbash M Transcriptional feedback and definition of the circadian pacemaker in *Drosophila* and animals Cold Spring Harb Symp Quant Biol 2007 72 75 84 10.1101/sqb.2007.72.062 18419264

[B83] Zheng X Sehgal A Probing the relative importance of molecular oscillations in the circadian clock Genetics 2008 178 1147 1155 10.1534/genetics.107.088658 18385110 PMC2278066

[B84] Yang Z Sehgal A Role of molecular oscillations in generating behavioral rhythms in *Drosophila* Neuron 2001 29 453 467 10.1016/S0896-6273(01)00218-5 11239435

[B85] Reppert SM Weaver DR Molecular analysis of mammalian circadian rhythms Annu Rev Physiol 2001 63 647 676 10.1146/annurev.physiol.63.1.647 11181971

[B86] Chen CH Dunlap JC Loros JJ *Neurospora* illuminates fungal photoreception Fungal Genet Biol 2010 47 922 929 10.1016/j.fgb.2010.07.005 20637887 PMC3649881

[B87] Ueda HR System-level identification of transcriptional circuits underlying mammalian circadian clocks Nat Genet 2005 37 187 192 10.1038/ng1504 15665827

[B88] Kako K Ishida N The role of transcription factors in circadian gene expression Neurosci Res 1998 31 257 264 10.1016/S0168-0102(98)00054-6 9809584

[B89] Matsumoto A A functional genomics strategy reveals *clockwork orange* as a transcriptional regulator in the *Drosophila* circadian clock Genes Dev 2007 21 1687 1700 10.1101/gad.1552207 17578908 PMC1899476

[B90] Shotkoski F Morris AC James AA Ffrench-Constan RH Functional analysis of a mosquito γ-aminobutyric acid receptor gene promoter Gene 1996 168 127 133 10.1016/0378-1119(95)00756-3 8654932

[B91] McDonald MJ Rosbash M Emery P Wild-type circadian rhythmicity is dependent on closely spaced E Boxes in the *Drosophila timeless* promoter Mol Cell Biol 2001 21 1207 1217 10.1128/MCB.21.4.1207-1217.2001 11158307 PMC99574

[B92] So WV *Takeout*, a novel *Drosophila* gene under circadian clock transcriptional regulation Mol Cell Biol 2000 20 6935 6944 10.1128/MCB.20.18.6935-6944.2000 10958689 PMC88769

[B93] Montminy MR Sevarino KA Wagner JA Mandel G Goodman RH Identification of a cyclic-AMP-responsive element within the rat somatostatin gene Proc Natl Acad Sci USA 1986 83 6682 6686 10.1073/pnas.83.18.6682 2875459 PMC386573

[B94] Lin SC Lin MH Horváth P Reddy KL Storti RV PDP1, a novel *Drosophila* PAR domain bZIP transcription factor expressed in developing mesoderm, endoderm and ectoderm, is a transcriptional regulator of somatic muscle genes Development 1997 124 4685 4696 9409684 10.1242/dev.124.22.4685

[B95] Cyran SA *vrille*, *Pdp1*, and *dClock* form a second feedback loop in the *Drosophila* circadian clock Cell 2003 112 329 341 10.1016/S0092-8674(03)00074-6 12581523

[B96] Ribbands CR Moonlight and house-haunting habits of female anophelines in West Africa Bull Entomol Res 1946 36 395 417 10.1017/S0007485300024056 21015619

[B97] Charlwood JD Mating does not affect the biting behaviour of *Anopheles gambiae* from the islands of São Tomé and Principe, West Africa Ann Trop Med Parasitol 2003 97 751 756 10.1179/000349803225002345 14613634

[B98] Molineaux L The Garki project: Research on the epidemiology and control of malaria in the Sudan savanna of West Africa 1980 World Health Organization

[B99] Mathenge EM Effect of permethrin-impregnated nets on exiting behavior, blood feeding success, and time of feeding of malaria mosquitoes (Diptera: Culicidae) in western Kenya J Med Entomol 2001 38 531 536 10.1603/0022-2585-38.4.531 11476333

[B100] Haddow AJ Ssenkubuge Y The mosquitoes of Bwamba County, Uganda. IX. Further studies on the biting behaviour of an outdoor population of the *Anopheles gambiae Giles* complex Bull Entomol Res 1973 62 407 414 10.1017/S0007485300003928

[B101] Taylor B Jones MDR The circadian rhythm of flight activity in the mosquito *Aedes aegypti* (L.): The phase-setting effects of light-on and light-off J Exp Biol 1969 51 59 70 5387705 10.1242/jeb.51.1.59

[B102] Brown MR Endogenous regulation of mosquito host-seeking behavior by a neuropeptide J Insect Physiol 1994 40 399 406 10.1016/0022-1910(94)90158-9

[B103] Huang DW Sherman BT Lempicki RA Systematic and integrative analysis of large gene lists using DAVID bioinformatics resources Nat Protoc 2009 4 44 57 19131956 10.1038/nprot.2008.211

[B104] Dennis G DAVID: Database for annotation, visualization, and integrated discovery Genome Biol 2003 4 R60 10.1186/gb-2003-4-9-r60 PMC193660 12734009

[B105] Dardente H Daily and circadian expression of neuropeptides in the suprachiasmatic nuclei of nocturnal and diurnal rodents Mol Brain Res 2004 124 143 151 10.1016/j.molbrainres.2004.01.010 15135222

[B106] Nunez AA Bult A McElhinny TL Smale L Daily rhythms of Fos expression in hypothalamic targets of the suprachiasmatic nucleus in diurnal and nocturnal rodents J Biol Rhythms 1999 14 300 306 10.1177/074873099129000713 10447310

[B107] Martínez GS Smale L Nunez AA Diurnal and nocturnal rodents show rhythms in orexinergic neurons Brain Res 2002 955 1 7 10.1016/S0006-8993(02)03264-X 12419515

[B108] Vosko AM Hagenauer MH Hummer DL Lee TM Period gene expression in the diurnal degu (*Octodon degus*) differs from the nocturnal laboratory rat (*Rattus norvegicus*) Am J Physiol Regul Integr Comp Physiol 2009 296 R353 R361 19036829 10.1152/ajpregu.90392.2008 PMC3834001

[B109] Patrick ML Aimanova K Sanders HR Gill SS P-type Na^+^/K^+^-ATPase and V-type H^+^-ATPase expression patterns in the osmoregulatory organs of larval and adult mosquito *Aedes aegypti* J Exp Biol 2006 209 4638 4651 10.1242/jeb.02551 17114398

[B110] Calvo E Dao A Pham VM Ribeiro JMC An insight into the sialome of *Anopheles funestus* reveals an emerging pattern in anopheline salivary protein families Insect Biochem Mol Biol 2007 37 164 175 10.1016/j.ibmb.2006.11.005 17244545 PMC1853278

[B111] Klein U Zimmermann B The vacuolar-type ATPase from insect plasma membrane: Immunocytochemical localization in insect sensilla Cell Tissue Res 1991 266 265 273 10.1007/BF00318182

[B112] Sessions OM Discovery of insect and human dengue virus host factors Nature 2009 458 1047 1050 10.1038/nature07967 19396146 PMC3462662

[B113] Nawa M Effects of bafilomycin A1 on Japanese encephalitis virus in C6/36 mosquito cells Arch Virol 1998 143 1555 1568 10.1007/s007050050398 9739334

[B114] Cociancich SO Park SS Fidock DA Shahabuddin M Vesicular ATPase-overexpressing cells determine the distribution of malaria parasite oocysts on the midguts of mosquitoes J Biol Chem 1999 274 12650 12655 10.1074/jbc.274.18.12650 10212245

[B115] Beyenbach KW Wieczorek H The V-type H^+^ ATPase: Molecular structure and function, physiological roles and regulation J Exp Biol 2006 209 577 589 10.1242/jeb.02014 16449553

[B116] Moriyama Y Maeda M Futai M The role of V-ATPase in neuronal and endocrine systems J Exp Biol 1992 172 171 178 1362770 10.1242/jeb.172.1.171

[B117] Hiesinger PR The v-ATPase V_0_ subunit a1 is required for a late step in synaptic vesicle exocytosis in *Drosophila* Cell 2005 121 607 620 10.1016/j.cell.2005.03.012 15907473 PMC3351201

[B118] Williamson WR Wang D Haberman AS Hiesinger PR A dual function of V_0_-ATPase a1 provides an endolysosomal degradation mechanism in *Drosophila melanogaster* photoreceptors J Cell Biol 2010 189 885 899 10.1083/jcb.201003062 20513768 PMC2878941

[B119] Pyza E Borycz J Giebultowicz JM Meinertzhagen IA Involvement of V-ATPase in the regulation of cell size in the fly's visual system J Insect Physiol 2004 50 985 994 10.1016/j.jinsphys.2004.08.003 15607501

[B120] Zuker CS The biology of vision of *Drosophila* Proc Natl Acad Sci USA 1996 93 571 576 10.1073/pnas.93.2.571 8570597 PMC40093

[B121] Katz B Minke B *Drosophila* photoreceptors and signaling mechanisms Front Cell Neurosci 2009 3 2 19623243 10.3389/neuro.03.002.2009 PMC2701675

[B122] Han J The fly CAMTA transcription factor potentiates deactivation of rhodopsin, a G protein-coupled light receptor Cell 2006 127 847 858 10.1016/j.cell.2006.09.030 17110341

[B123] Colley NJ Baker EK Stamnes MA Zuker CS The cyclophilin homolog ninaA is required in the secretory pathway Cell 1991 67 255 263 10.1016/0092-8674(91)90177-Z 1913822

[B124] Wang T Wang X Xie Q Montell C The SOCS box protein STOPS is required for phototransduction through its effects on phospholipase C Neuron 2008 57 56 68 10.1016/j.neuron.2007.11.020 18184564 PMC2253723

[B125] Fleissner G Fleissner G Efferent control of visual sensitivity in arthropod eyes: With emphasis on circadian rhythms 1987 Stuttgart, New York: G. Fischer Verlag

[B126] Yau KW Hardie RC Phototransduction motifs and variations Cell 2009 139 246 264 10.1016/j.cell.2009.09.029 19837030 PMC2885920

[B127] Zhou JJ He XL Pickett JA Field LM Identification of odorant-binding proteins of the yellow fever mosquito *Aedes aegypti*: Genome annotation and comparative analyses Insect Mol Biol 2008 17 147 163 10.1111/j.1365-2583.2007.00789.x 18353104

[B128] Benton R Sachse S Michnick SW Vosshall LB Atypical membrane topology and heteromeric function of *Drosophila* odorant receptors *in vivo* PLoS Biol 2006 4 e20 10.1371/journal.pbio.0040020 16402857 PMC1334387

[B129] Wise de Valdez MR Genetic elimination of dengue vector mosquitoes Proc Natl Acad Sci USA 2011 108 4772 4775 10.1073/pnas.1019295108 21383140 PMC3064365

[B130] Meredith JM Site-specific integration and expression of an anti-malarial gene in transgenic *Anopheles gambiae* significantly reduces *Plasmodium* infections PLoS One 2011 6 e14587 10.1371/journal.pone.0014587 21283619 PMC3026776

[B131] Matsumoto A *Period* gene of *Bactrocera cucurbitae* (Diptera: Tephritidae) among strains with different mating times and sterile insect technique Ann Entomol Soc Am 2008 101 1121 1130 10.1603/0013-8746-101.6.1121

[B132] Fuchikawa T The clock gene *cryptochrome* of *Bactrocera cucurbitae* (Diptera: Tephritidae) in strains with different mating times Heredity 2010 104 387 392 10.1038/hdy.2009.167 20010960

[B133] Mbogo CNM Baya NM Ofulla AVO Githure JI Snow RW The impact of permethrin-impregnated bednets on malaria vectors of the Kenyan coast Med Vet Entomol 1996 10 251 259 10.1111/j.1365-2915.1996.tb00739.x 8887336

[B134] Lawson D VectorBase: A data resource for invertebrate vector genomics Nucleic Acids Res 2009 37 D583 D587 10.1093/nar/gkn857 19028744 PMC2686483

[B135] Xi Z Ramirez JL Dimopoulos G The *Aedes aegypti* toll pathway controls dengue virus infection PLoS Pathog 2008 4 e1000098 10.1371/journal.ppat.1000098 18604274 PMC2435278

[B136] Cooley JW Tukey JW An algorithm for the machine calculation of complex Fourier series Math Computation 1965 19 297 301 10.1090/S0025-5718-1965-0178586-1

[B137] Cook C Bernfeld M CH 2. Matched Filter Radar signals: An introduction to theory and application 1993 Boston: Artech House

[B138] Hunter S InterPro: The integrative protein signature database Nucleic Acids Res 2009 37 D211 D215 10.1093/nar/gkn785 18940856 PMC2686546

[B139] Eisen MB Spellman PT Brown PO Botstein D Cluster analysis and display of genome-wide expression patterns Proc Natl Acad Sci USA 1998 95 14863 14868 10.1073/pnas.95.25.14863 9843981 PMC24541

[B140] Saldanha AJ Java Treeview–extensible visualization of microarray data Bioinformatics 2004 20 3246 3248 10.1093/bioinformatics/bth349 15180930

[B141] Dana AN Gene expression patterns associated with blood-feeding in the malaria mosquito *Anopheles gambiae* BMC Genomics 2005 6 5 10.1186/1471-2164-6-5 15651988 PMC546002

[B142] Müller P Donnelly MJ Ranson H Transcription profiling of a recently colonised pyrethroid resistant *Anopheles gambiae* strain from Ghana BMC Genomics 2007 8 36 10.1186/1471-2164-8-36 17261191 PMC1797171

[B143] Biessmann H The *Anopheles gambiae* Odorant Binding Protein 1 (AgamOBP1) mediates indole recognition in the antennae of female mosquitoes PLoS One 2010 5 e9471 10.1371/journal.pone.0009471 20208991 PMC2830424

