# Supplementary material for: Extensive circadian and light regulation of the transcriptome in the malaria mosquito Anopheles gambiae
Source: BMC Genomics. 2013 Apr 3;14:218. doi: 10.1186/1471-2164-14-218 (PMC3642039; doi:10.1186/1471-2164-14-218)
Supplement: Additional file 1 — Rhythmic An. gambiae probes, by statistical test cutoff value. Only probes with a mean fluorescent intensity >20 across the time course were analyzed. Probes indicated as rhythmic using COSOPT or DFT were found rhythmic in both of the two replicate runs. In JTK_CYCLE and COSOPT, only probes where period length under LD conditions was between 20 hr to 28 hr or in DD conditions between 18.5 hr - 26.5 hr are reported. Note DFT performed on 24 hr signal for all runs, see methods for more details. [file 1471-2164-14-218-S1.docx]

|  | COSOPT | |  | JTK_CYCLE | |  | DFT | |
| --- | --- | --- | --- | --- | --- | --- | --- | --- |
| LD Heads | p < 0.2 | 2338 |  | q < 0.1 | 2236 |  | s > 0.3 | 2560 |
|  | p < 0.15 | 2114 |  | q < 0.05 | 1943 |  | s > 0.35 | 1776 |
|  | p < 0.1 | 1819 |  | q < 0.01 | 1404 |  | s > 0.4 | 1106 |
|  | p < 0.05 | 1220 |  | q < 0.001 | 892 |  | s> 0.45 | 618 |
|  | p < 0.01 | 81 |  | q< 0.0001 | 535 |  | s > 0.5 | 295 |
|  |  |  |  |  |  |  |  |  |
| LD Bodies | p < 0.2 | 1146 |  | q < 0.1 | 1428 |  | s > 0.3 | 1438 |
|  | p < 0.15 | 988 |  | q < 0.05 | 1216 |  | s > 0.35 | 807 |
|  | p < 0.1 | 782 |  | q < 0.01 | 767 |  | s > 0.4 | 392 |
|  | p < 0.05 | 443 |  | q < 0.001 | 417 |  | s > 0.45 | 156 |
|  | p < 0.01 | 12 |  | q< 0.0001 | 187 |  | s > 0.5 | 40 |
|  |  |  |  |  |  |  |  |  |
| DD Heads | p < 0.2 | 1738 |  | q < 0.1 | 1196 |  | s > 0.3 | 789 |
|  | p < 0.15 | 1462 |  | q < 0.05 | 913 |  | s > 0.35 | 331 |
|  | p < 0.1 | 1107 |  | q < 0.01 | 495 |  | s > 0.4 | 133 |
|  | p < 0.05 | 623 |  | q < 0.001 | 247 |  | s > 0.45 | 36 |
|  | p < 0.01 | 46 |  | q< 0.0001 | 134 |  | s > 0.5 | 5 |
|  |  |  |  |  |  |  |  |  |
| DD Bodies | p < 0.2 | 987 |  | q < 0.1 | 580 |  | s > 0.3 | 786 |
|  | p < 0.15 | 789 |  | q < 0.05 | 458 |  | s > 0.35 | 344 |
|  | p < 0.1 | 584 |  | q < 0.01 | 277 |  | s > 0.4 | 155 |
|  | p < 0.05 | 316 |  | q < 0.001 | 141 |  | s > 0.45 | 60 |
|  | p < 0.01 | 9 |  | q< 0.0001 | 71 |  | s > 0.5 | 18 |
