# Supplementary figures and images for: Extensive circadian and light regulation of the transcriptome in the malaria mosquito Anopheles gambiae
Source: BMC Genomics. 2013 Apr 3;14:218. doi: 10.1186/1471-2164-14-218 (PMC3642039; doi:10.1186/1471-2164-14-218)

LD Body JTK\_CYCLE  $q < 0.1$

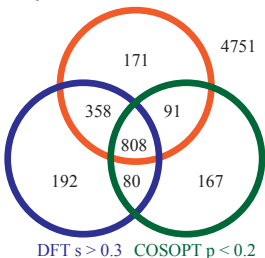

DD Head JTK\_CYCLE  $q < 0.1$

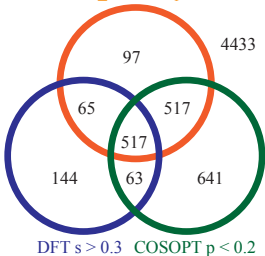

DD Body JTK\_CYCLE  $q < 0.1$

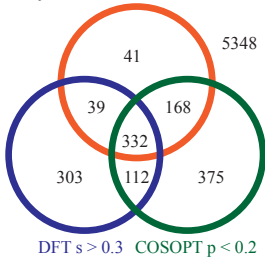

Supplement: Additional file 2 — Analysis of expression data by various algorithms reveals overlap in An. gambiae probes deemed rhythmic. Venn diagrams show the number of probes in LD bodies and DD heads and bodies identified as rhythmic using the JTK_CYCLE, DFT and COSOPT algorithms at the statistical cutoffs indicated. In LD bodies, a total of 808 probes were identified as rhythmic using all three algorithms, representing 148 new rhythmic probes from those identified previously [30]. In DD heads, a total of 517 probes were found rhythmic using all three conditions (47 new probes). In DD bodies, a total of 332 probes were identified as rhythmic using all three algorithms (32 new probes). Note DFT analysis limits the number of probes that may be deemed rhythmic under DD conditions; see methods for more information. See Figure 1 for LD head Venn diagram. See Additional file 3 for list of probes newly identified as rhythmic. The numbers outside the Venn diagrams represent the number of probes with a mean fluorescent intensity above background that were not scored as rhythmic by any of the algorithms. [file 1471-2164-14-218-S2.pdf]

A.

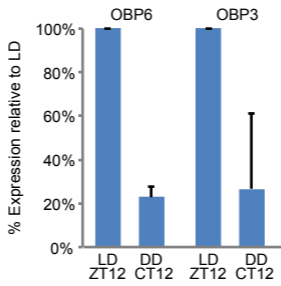

B.

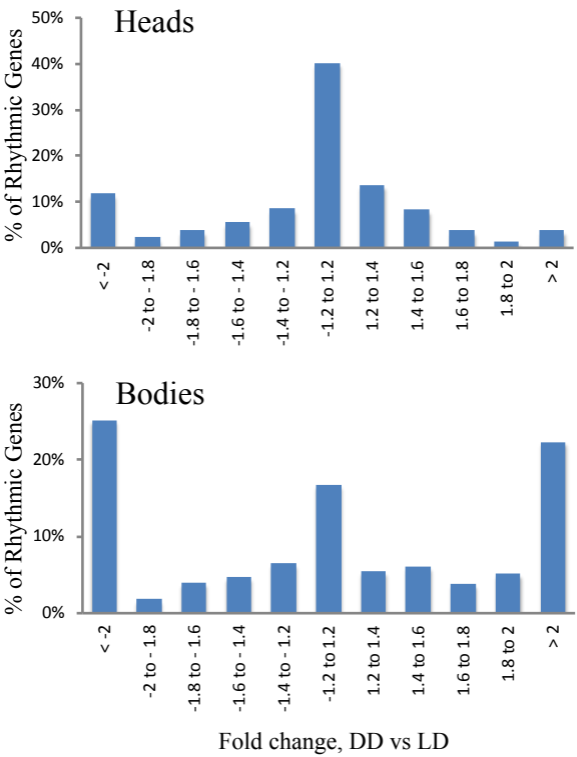

Supplement: Additional file 4 — An. gambiae gene expression changes in LD versus DD conditions. (A) qRT-PCR confirmation of reduction in expression in OBP3 and OBP6 under DD versus LD conditions. Values are mean ± SD of gene expression as a percentage normalized to the LD value of 100%. Female mated, non-blood fed mosquitoes, 5-7 day post emergence from mosquitoes reared concurrently under different lighting conditions were collected under LD conditions or DD conditions (from mosquitoes placed in darkness for 24 hr) at ZT/CT 12. (B) Average gene expression changes between LD and DD conditions as measured by microarray analysis across 44 hr. The average expression level averaged across all 12 time points was analyzed in both LD and DD, and the fold change difference in expression level between LD and DD determined. Most probes showed similar expression levels between LD and DD. However, significant variation occured in a subset of genes. This was especially pronounced in bodies, where 47% of the rhythmic genes had >2 fold difference in expression levels between LD and DD conditions. [file 1471-2164-14-218-S4.pdf]
