# Supplementary material for: Extensive circadian and light regulation of the transcriptome in the malaria mosquito Anopheles gambiae
Source: BMC Genomics. 2013 Apr 3;14:218. doi: 10.1186/1471-2164-14-218 (PMC3642039; doi:10.1186/1471-2164-14-218)
Supplement: Additional file 5 — Amplitude measures for An. gambiae clock genes expressed in the head under LD and DD conditions. Amplitudes calculated as peak divided by nadir normalized fluorescence values and where peak-to-nadir occurred with an interval of 8-16 hr. The JTK_CYCLE amplitude value reflects the 1-cycle median sign-adjusted deviation from the median in relation to the optimal cosine pattern. (DOCX 16 kb) [file 1471-2164-14-218-S5.docx]

|  |  |  | **LD Amplitude** | | **DD Amplitude** | | **JTK Amplitude** | |
| --- | --- | --- | --- | --- | --- | --- | --- | --- |
| **Symbol** | **Gene ID** | **Probe ID** | **Cycle 1** | **Cycle 2** | **Cycle 1** | **Cycle 1** | **LD** | **DD** |
| *CRY2* | AGAP004261 | Ag.UNKN.314.0_CDS_at | 81 ± 2.1 | 81.6 ± 2.9 | 24.3 ± 8.8 | 14.7 ± 4.2 | 2.23 | 1.23 |
| *CRY2* | AGAP004261 | Ag.2R.2952.1_a_at | 6.6 ± 3.1 | 6.1 ± 3.0 | 1.9 ± 0.6 | 1.4 ± 0.2 | 0.60 | 0.29 |
| *CRY2* | AGAP004261 | Ag.2R.2952.0_CDS_at | 1.9 ± 0.0 | 2.8 ± 1.7 | fail | fail | 0.12 | 0.07 |
| *PER* | AGAP001856 | Ag.2R.2131.0_CDS_at | 45.5 ± 13.7 | 39.5 ± 11.2 | 8 ± 1.8 | 8.1 ± 0.1 | 2.61 | 1.13 |
| *TIM* | AGAP008288 | Ag.3R.272.0_CDS_a_at | 5.6 ± 0.3 | 5.8 ± 0.3 | 4.1 ± 0.6 | 3.9 ± 0.8 | 1.08 | 1.04 |
| *TIM* | AGAP008288 | Ag.3R.272.0_UTR_a_at | 6.0 ± 0.3 | 5.5 ± 0.1 | 4 ± 0.4 | 3.5 ± 0.8 | 1.38 | 1.10 |
| *CYC* | AGAP005655 | Ag.2L.643.0_CDS_a_at | 10.2 ± 1.4 | 8.2 ± 1.9 | 8 ± 0.7 | 6.4 ± 0.6 | 0.86 | 1.45 |
| *CYC* | AGAP005655 | Ag.2L.643.1_CDS_a_at | 24.2 ± 0.6 | 20.5 ± 1.8 | 24.4 ± 6.7 | 6.1 ± 1.4 | 2.47 | 1.39 |
| *PDP1* | AGAP006376 | Ag.2L.904.0_CDS_at | 3.5 ± 0.0 | 4.2 ± 0.7 | 2.5 ± 0.4 | 3 ± 0.3 | 1.18 | 0.33 |
