# Supplementary material for: Extensive circadian and light regulation of the transcriptome in the malaria mosquito Anopheles gambiae
Source: BMC Genomics. 2013 Apr 3;14:218. doi: 10.1186/1471-2164-14-218 (PMC3642039; doi:10.1186/1471-2164-14-218)
Supplement: Additional file 6 — Promoter sequence search of light- and circadian- driven gene expression. Specific promoter search criteria and the results of searching for defined response elements [49,88-95] in the 5kb 5' region upstream of the transcription start site of type I OBPs, type II OBPs and the other genes found clustering with those OBPs (see Figure 3), and type III OBPs. The table provides the gene name, VectorBase ID and the number and identity of consensus sequences found in the 1kb and 5kb upstream region of the genes. For some genes, the full 5kb region was not available, as it would overlap with the predicted coding region of another gene. In such cases, only the region that did not overlap was considered; the number of base pairs considered is provided in the “Upstream region (bp)” column. [file 1471-2164-14-218-S6.docx]

**Promoter search criteria**

**In general, we utilized the promoter search criteria of Claridge-Chang *et al*. (1).**

**Specifically:**

**Generic E box:** CANNTG (2, 3)

**Stringent E box:** CANGTG or CACNTG

**Canonical circadian E box:** CACGTG with a perfect match (4)

**W box:** TTGGCCAGCAA with two mismatches (4, 5)

**CRE (Ca^2+^/cAMP response element):** TGACGTCA with one mismatch (6)

**PERR (Per repeat):** GTTCGCACAA with one mismatch (4)

**TER (Tim-E-box-like repeat):** GCACGTTG with one mismatch (4)

**PDP1 (PDP1 binding site):** GAATTTTGTAAC with two mismatches (7, 8)

**TYPE I**

| **Gene** | **Promoters Found (1000 bp)** | **Promoters Found (5000 bp)** | **Upstream region (bp)** |
| --- | --- | --- | --- |
| OBP6  AGAP003530 | W box  2 TER *  8 Generic E box  2 Stringent E box  2 Canonical circadian E box | W box  2 TER *  10 Generic E box  4 Stringent E box 3 Canonical circadian E box | 1286 |
| OBP7  AGAP001556 | 13 Generic E box 2 Stringent E box | 2 TER  1 PDP1  38 Generic E box  7 Stringent E box | 3275 |
| OBP26  AGAP012321 | TER  4 Generic E box  1 Stringent E box | 2 CRE  TER  30 Generic E box  3 Stringent E box | 2903 |
| OBP14  AGAP002905 | PDP1  12 Generic E box  6 Stringent E box  2 Canonical circadian E box | PDP1  12 Generic E box  6 Stringent E box 2 Canonical circadian E box | 899 |

**TYPE II**

| **Gene** | **Promoters Found (1000 bp)** | **Promoters Found (5000 bp)** | **Upstream region (bp)** |
| --- | --- | --- | --- |
| OBP2  AGAP003306 | W box  CRE  TER  7 Generic E box  2 Stringent E box  Canonical circadian E box | 2 W box  4 CRE  7 TER *  PDP1  38 Generic E box  12 Stringent E box  8 Canonical circadian E box | 5000 |
| OBP3  AGAP001409 | TER  11 Generic E box  1 Stringent E box | 2 CRE  6 TER  37 Generic E box  5 Stringent E box  2 Canonical circadian E box | 5000 |
| OBP4  AGAP010489 | PERR  5 Generic E box  2 Stringent E box | 2 W box  PERR  TER  2 PDP1  48 Generic E box  10 Stringent E box | 5000 |
| OBP5  AGAP009629 | TER *  PDP1  6 Generic E box  Stringent E box  Canonical circadian E box | W box  9 TER *  2 PDP1  52 Generic E box  6 Stringent E box 16 Canonical circadian E box | 5000 |
| OBP17  AGAP003309 | 4 Generic E box  Stringent E box Canonical circadian E box | 4 CRE  2 TER  18 Generic E box  5 Stringent E box  2 Canonical circadian E box | 5000 |
| OBP22  AGAP010409 | W box  TER  PDP1  12 Generic E box  Stringent E box | W box  2 TER  PDP1  24 Generic E box  Stringent E box | 2019 |
| Interpro: peptidase  AGAP000994 | PERR  2 TER  8 Generic E box | PERR  2 TER  20 Generic E box 3 Stringent E box | 3154 |
| Interpro: cellular retinaldehyde  AGAP005701 | TER  11 Generic E box  Stringent E box Canonical circadian E box | W box  3 TER  2 PDP1  52 Generic E box  8 Stringent E box 4 Canonical circadian E box | 5000 |
| GSTU3  AGAP009342 | W box  CRE  11 Generic E box | 2 W box  4 CRE  TER  58 Generic E box  6 Stringent E box 2 Canonical circadian E box | 5000 |
| AAEG: pxmp2  AGAP006040 | 12 Generic E box  3 Stringent E box | 3 W box  2 CRE  5 TER  PDP1  48 Generic E box  10 Stringent E box  2 Canonical circadian E box | 5000 |
| OBP20  AGAP005208 | 7 Generic E box  Stringent E box | W box  TER  30 Generic E box  4 Stringent E box | 4861 |
| Unknown  AGAP009056 | 2 TER  8 Generic E box | 2 TER  8 Generic E box | 820 |
| SNMP1  AGAP002451 | 2 TER  8 Generic E box 3 Stringent E box 2 Canonical circadian E box | 2 W box  2 CRE  PERR  8 TER *  66 Generic E box  19 Stringent E box 11 Canonical circadian E box | 5000 |

**TYPE III**

| **Gene** | **Promoters Found (1000 bp)** | **Promoters Found (5000 bp)** | **Upstream region (bp)** |
| --- | --- | --- | --- |
| OBP51  AGAP006077 | TER  3 Generic E box  Stringent E box Canonical circadian E box | W box  3 TER  6 Generic E box  2 Stringent E box 2 Canonical circadian E box | 5000 |
| OBP29  AGAP012331 | W box  CRE  2 TER  11 Generic E box | W box  2 CRE  3 TER  16 Generic E box  Stringent E box | 1525 |
| OBP47  AGAP007287 | CRE  3 TER *  13 Generic E box 2 Stringent E box 3 Canonical circadian E box | 2 CRE  5 TER *  18 Generic E box  4 Stringent E box 3 Canonical circadian E box | 1388 |
| Obp54  AGAP006080 | TER  4 Generic E box  3 Stringent E box 2 Canonical circadian E box | TER  4 Generic E box 3 Stringent E box 2 Canonical circadian E box | 552 |
| Obp57  AGAP011368 | 9 Generic E box  3 Stringent E box Canonical circadian E box | 18 Generic E box 4 Stringent E box  2 Canonical circadian E box | 1779 |

* Cases where some TER sequences are also identified as Canonical circadian E boxes.

**References**

1. Claridge-Chang A*, et al* (2001) Circadian regulation of gene expression systems in the *Drosophila* head*. Neuron* 32: 657-671.

2. Kako K & Ishida N (1998) The role of transcription factors in circadian gene expression*. Neurosci Res* 31: 257-264.

3. Matsumoto A*, et al* (2007) A functional genomics strategy reveals *clockwork orange* as a transcriptional regulator in the *Drosophila* circadian clock*. Genes Dev* 21: 1687-1700.

4. McDonald MJ, Rosbash M & Emery P (2001) Wild-type circadian rhythmicity is dependent on closely spaced E Boxes in the *Drosophila timeless* promoter*. Mol Cell Biol* 21: 1207-1217.

5. So WV*, et al* (2000) *Takeout*, a novel *Drosophila* gene under circadian clock transcriptional regulation*. Mol Cell Biol* 20: 6935-6944.

6. Montminy MR, Sevarino KA, Wagner JA, Mandel G & Goodman RH (1986) Identification of a cyclic-AMP-responsive element within the rat somatostatin gene*. Proc Natl Acad Sci USA* 83: 6682-6686.

7. Lin SC, Lin MH, Horváth P, Reddy KL & Storti RV (1997) PDP1, a novel *Drosophila* PAR domain bZIP transcription factor expressed in developing mesoderm, endoderm and ectoderm, is a transcriptional regulator of somatic muscle genes*. Development* 124: 4685-4696.

8. Cyran SA*, et al* (2003) *vrille*, *Pdp1*, and *dClock* form a second feedback loop in the *Drosophila* circadian clock*. Cell* 112: 329-341.
